# Beyond Transposons: *TIGD1* as a Pan-Cancer Biomarker and Immune Modulator

**DOI:** 10.3390/genes16060674

**Published:** 2025-05-30

**Authors:** Merve Gulsen Bal Albayrak, Tuğcan Korak, Gurler Akpinar, Murat Kasap

**Affiliations:** Department of Medical Biology, Faculty of Medicine, Kocaeli University, Kabaoglu District, Baki Komsuoglu Blvd. No:21, Umuttepe, 41001 Kocaeli, Turkey; tugcan.korak@kocaeli.edu.tr (T.K.); gurlerak@kocaeli.edu.tr (G.A.); mkasap@kocaeli.edu.tr (M.K.)

**Keywords:** *TIGD1*, pan-cancer analysis, immune microenvironment, drug resistance, cancer prognosis, immunotherapy, tumor mutational burden, microsatellite instability

## Abstract

**Background/Objectives**: *TIGD1* (Trigger Transposable Element Derived 1) is a recently identified oncogene with largely unexplored biological functions. Emerging evidence suggests its involvement in multiple cellular processes across cancer types. This study aimed to perform a comprehensive pan-cancer analysis of *TIGD1* to evaluate its expression patterns, diagnostic utility, prognostic value, and association with immunotherapy response and drug resistance. **Methods**: Transcriptomic and clinical data from TCGA and GTEx were analyzed using various bioinformatic tools. Expression profiling, survival analysis, immune correlation studies, gene set enrichment, single-cell sequencing, and drug sensitivity assessments were performed. **Results**: *TIGD1* was found to be significantly upregulated in various tumor types, with notably high expression in colon adenocarcinoma. Elevated *TIGD1* expression was associated with poor prognosis in several cancers. *TIGD1* levels correlated with key features of the tumor immune microenvironment, including immune checkpoint gene expression, TMB, and MSI, suggesting a role in modulating anti-tumor immunity. GSEA and single-cell analyses implicated *TIGD1* in oncogenic signaling pathways. Furthermore, high *TIGD1* expression was linked to resistance to several therapeutic agents, including Zoledronate, Dasatinib, and BLU-667. **Conclusions**: *TIGD1* may serve as a promising diagnostic and prognostic biomarker, particularly in colon, gastric, liver, and lung cancers. Its strong associations with immune modulation and therapy resistance highlight its potential as a novel target for precision oncology and immunotherapeutic intervention.

## 1. Introduction

Cancer remains a major global health burden, necessitating the discovery of novel biomarkers for early detection, accurate prognosis, and effective treatment strategies [1,2]. Among the emerging areas of oncological research, transposable elements (TEs)—also known as mobile genetic elements—have garnered increasing attention due to their roles in genomic instability, epigenetic modulation, and immune regulation [3,4,5,6]. TEs constitute nearly half of the human genome and, although typically silenced by host epigenetic mechanisms, their aberrant reactivation in cancer can contribute to oncogenesis through insertional mutagenesis, dysregulated gene expression, and activation of immune evasion pathways [7].

In addition to their genomic impact, TE-derived transcripts can mimic viral sequences and stimulate innate immune responses via pattern recognition receptors (PRRs), thereby reshaping the tumor immune microenvironment. These immunomodulatory properties position certain TEs as potential modulators of immunotherapy response, particularly immune checkpoint blockade [8,9]. Among the genes derived from ancestral TEs, *TIGD1* (Tigger Transposable Element Derived 1) is of particular interest. Unlike other well-characterized TE-derived genes that have established functions in host development or immunity, *TIGD1* is a relatively uncharacterized member of the Tigger TE family. Its evolutionary conservation, consistent overexpression in several cancer types, and emerging correlations with immunological and clinical parameters underscore its potential functional relevance in cancer biology [10,11].

Despite emerging evidence implicating *TIGD1* in oncogenesis, its role in cancer remains largely elusive. Preliminary studies have reported *TIGD1* overexpression in colorectal, gastric, and lung cancers, where it correlates with poor prognosis and increased immune cell infiltration [12]. Furthermore, recent findings suggest that *TIGD1* may also contribute to immune regulation and drug resistance [13]. However, these observations are limited to a handful of cancer types, and its pan-cancer significance, underlying regulatory mechanisms, and associations with immunological or therapeutic features have not been systematically explored. Therefore, a comprehensive investigation is warranted to evaluate *TIGD1* expression across diverse malignancies, assess its diagnostic and prognostic value, and elucidate its mechanistic role in tumor progression and therapeutic resistance.

To address these gaps, this study investigates *TIGD1* expression across multiple cancers to determine its oncogenic potential. Additionally, it evaluates the prognostic and diagnostic significance of *TIGD1* across different tumor types while exploring its role in immune regulation, particularly its interactions with immune checkpoint molecules, tumor mutational burden (TMB, the total number of mutations per megabase of tumor DNA), and microsatellite instability (MSI, a condition of genetic hypermutability resulting from impaired DNA mismatch repair). Furthermore, this study examines the association between *TIGD1* and drug sensitivity, identifying potential links to chemoresistance. By integrating multi-omics datasets, we seek to establish *TIGD1* as a candidate biomarker in cancer biology and explore its potential as a therapeutic target. This study provides a comprehensive, pan-cancer perspective on *TIGD1*, offering insights into its role in tumor progression, immune interactions, and response to treatment.

**Figure 1:**
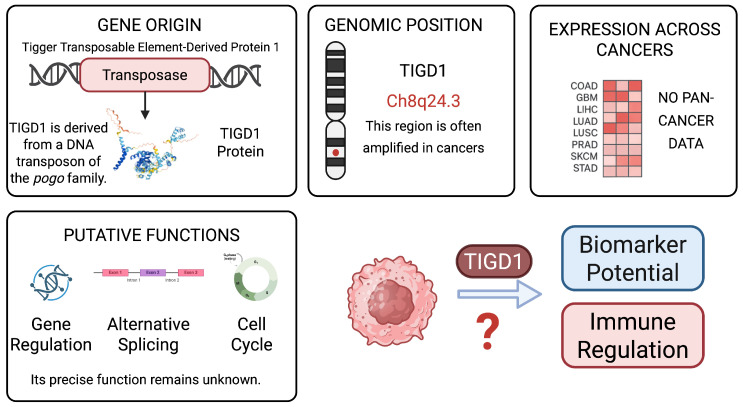
Overview of TIGD1 as a transposable element-derived gene of oncogenic interest. This schematic summarizes TIGD1’s origin from the pogo DNA transposon family, its chromosomal location at 8q24.3 (a region frequently amplified in cancer), and proposed functional roles including gene regulation, alternative splicing, and cell cycle regulation. Despite lacking precise functional characterization, TIGD1 shows expression across various cancers and has not been comprehensively studied in a pan-cancer context. These features suggest its potential involvement in tumor biology, particularly in biomarker discovery and immune modulation.

To provide a visual overview of TIGD1’s transposable element origin, genomic context, expression patterns, and rationale for selection, we present a schematic summary in Figure 1.

## 2. Materials and Methods

### 2.1. Expression Analysis of TIGD1 Pan-Cancer

*TIGD1* gene expression levels across TCGA tumors were analyzed using publicly available databases, including TIMER2.0 and GEPIA2.0, with statistical significance assessed via the Wilcoxon test [14]. For cancer types lacking normal tissue controls in TCGA, corresponding normal tissue data were retrieved from the GTEx dataset. The relationship between *TIGD1* expression and tumor staging was evaluated using the pathological stage module of GEPIA2.0 [15], while its correlation with cancer subtypes was examined via TISIDB [16].

### 2.2. Diagnostic Analysis of TIGD1

To analyze the diagnostic significance of the *TIGD1* gene across various cancers, receiver operating characteristic (ROC) curves were generated using the TCGA and GTEx data from the UCSC Xena database [17]. Subsequently, the ROC curves were analyzed using MedCalc (version 22.016; RRID:SCR_015044) software to calculate the area under the curve (AUC). AUC values between 0.5 and 0.6 were considered to have “no diagnostic value”; 0.6–0.75, “moderate diagnostic value”; and 0.75–1.0, “high diagnostic value” [18].

### 2.3. Prognostic Analysis of TIGD1

Cox regression analyses were undertaken to evaluate the prognostic significance of *TIGD1* in predicting overall survival (OS) and disease-free survival (DFS) across various cancers. Survival analyses were visually represented through Kaplan–Meier plots, generated using the KM plotter tool for both OS and DFS endpoints. Significance was established at a threshold of *p* < 0.05 [19].

### 2.4. Immune Correlation Analysis

The association between *TIGD1* expression and tumor immune characteristics was explored using multiple bioinformatics tools. TISIDB was employed to evaluate correlations with molecular and immune subtypes, while immune cell infiltration levels were analyzed using CIBERSORT. ESTIMATE scores were computed to determine the association between *TIGD1* expression and the tumor microenvironment (TME), including stromal and immune components. The relationships between *TIGD1* expression, tumor mutational burden, microsatellite instability, and immune checkpoint gene expression were investigated using Sangerbox 3.0.

### 2.5. Gene Set Enrichment and Single-Cell Sequencing Data Analysis

Gene set enrichment analysis (GSEA) was conducted using the LinkedOmics database to identify biological pathways associated with TIGD1 expression across cancers [20]. Single-cell RNA sequencing data were obtained from CancerSEA to examine TIGD1’s correlation with functional cancer states [21]. The potential role of TIGD1 in drug sensitivity was assessed using RNA-seq and drug response data from the NCI-60 cell line panel via CellMiner [22]. Statistical analyses were performed using IBM SPSS 20.0, with significance set at *p* < 0.05.

### 2.6. Drug Sensitivity Analysis

The potential role of *TIGD1* in drug sensitivity was assessed using RNA-seq and drug response data from the NCI-60 cell line panel via CellMiner. Statistical analyses were performed using IBM SPSS 20.0, with significance set at *p* < 0.05.

## 3. Results

### 3.1. Pan-Cancer TIGD1 Gene Expression Analysis

**Figure 2:**
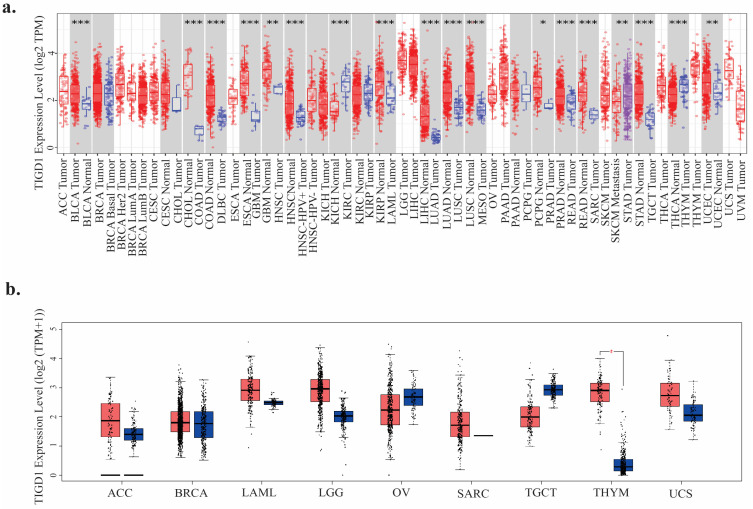
Pan-cancer *TIGD1* mRNA expression. (**a**) *TIGD1* expression across tumor and normal tissues from TCGA and GTEx via TIMER2.0 (* *p* < 0.05; ** *p* < 0.01; *** *p* < 0.001). (**b**) *TIGD1* expression in cancers lacking normal data in TIMER, analyzed via GEPIA (*p* < 0.001). TPM = transcripts per million. Tumor, normal, and metastasis samples are shown in red, blue, and purple, respectively.

To assess *TIGD1* gene expression across TCGA tumors, we utilized the TIMER2.0 database, revealing significant differences between cancer and normal tissues in BLCA, CHOL, ESCA, GBM, HNSC, KICH, KIRP, LIHC, LUAD, LUSC, PCPG, PRAD, READ, SKCM, STAD, THCA, and UCEC. While *TIGD1* expression was generally higher in tumors, KICH and THCA exhibited higher expression in normal tissues (Figure 2a). Since normal tissue controls were absent for some cancers, additional expression data were integrated from the GTEx project, identifying distinct expression patterns in adrenocortical carcinoma (ACC), acute myeloid leukemia (LAML), brain lower-grade glioma (LGG), thymoma (THYM), and uterine carcinosarcoma (UCS), with significant alterations observed only in THYM (Figure 2b).

**Figure 3:**
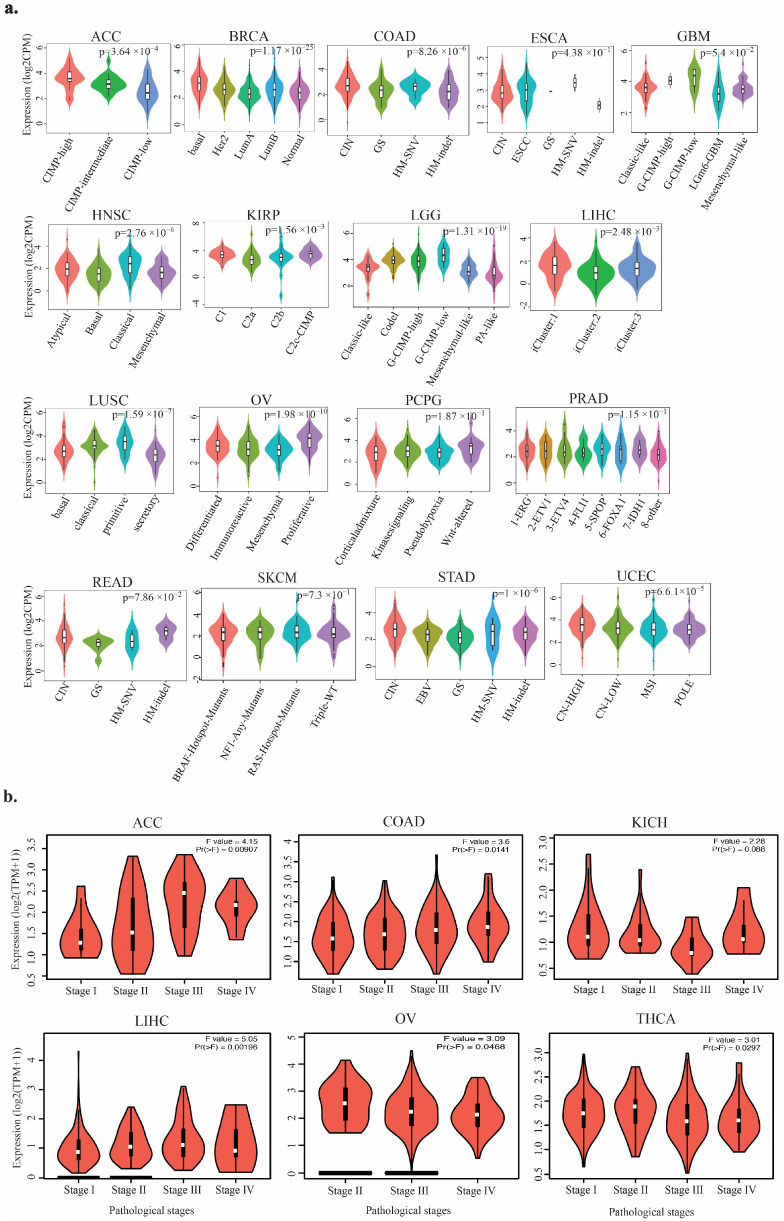
Correlation of *TIGD1* expression with tumor subtypes and pathological stages. (**a**) Violin plots depicting TIGD1 mRNA expression across molecular subtypes of various tumors, as obtained from TISIDB. Expression values are shown as counts per million (CPM). (**b**) Violin plots illustrating TIGD1 expression across pathological stages (stages I–IV) in adrenocortical carcinoma (ACC), colon adenocarcinoma (COAD), kidney chromophobe carcinoma (KICH), liver hepatocellular carcinoma (LIHC), ovarian serous cystadenocarcinoma (OV), and thyroid carcinoma (THCA). Expression values are represented as Log2(TPM + 1), and statistical significance was assessed using the Kruskal–Wallis test (*p* < 0.05).

Further analysis using TISIDB demonstrated that *TIGD1* expression was significantly associated with the molecular subtypes of 11 out of 17 tumors, including ACC, BRCA, COAD, HNSC, KIRP, LGG, LIHC, LUSC, OV, STAD, and UCEC (Figure 3a). Additionally, the pathological staging analysis via GEPIA2.0 indicated that *TIGD1* expression varied significantly across different cancer stages in ACC, COAD, KICH, LIHC, OV, and THCA (Figure 3b), supporting its potential role in tumor progression.

### 3.2. Diagnostic Analysis—ROC

**Figure 4:**
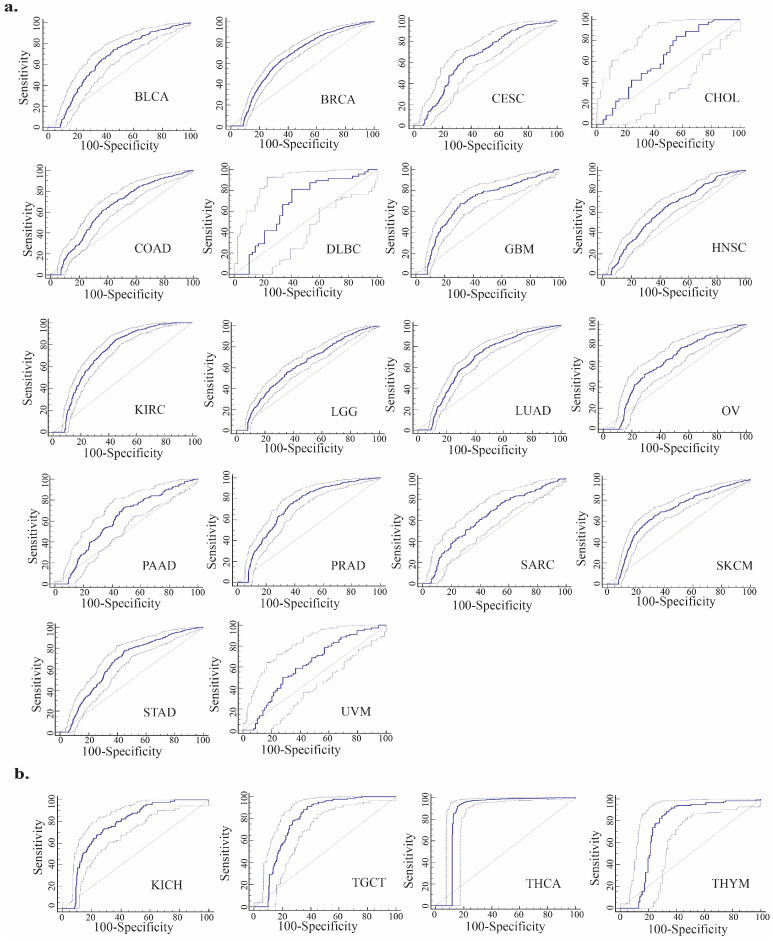
Diagnostic value of TIGD1 in pan-cancer. (**a**) Receiver operating characteristic (ROC) curves illustrating cancer types where TIGD1 shows moderate diagnostic performance (area under the curve [AUC] between 0.60 and 0.75; *p* < 0.05). (**b**) ROC curves for cancer types demonstrating high diagnostic accuracy of TIGD1 (AUC between 0.75 and 1.00; *p* < 0.05). ROC curves are represented by solid dark blue lines and corresponding 95% confidence intervals (CIs) are shown as light blue dashed lines.

ROC analyses were conducted to assess the diagnostic significance of TIGD1 expression across pan-cancer through calculating the AUC values (Table S1). Among the 32 tumors analyzed, 22 exhibited diagnostic value (*p* < 0.05), with four showing high diagnostic value and the remainder demonstrating moderate diagnostic significance. Notably, TIGD1 expression emerged as a promising diagnostic marker in kidney chromophobe (KICH; AUC = 0.770), testicular germ cell tumors (TGCTs; AUC = 0.783), thyroid carcinoma (THCA; AUC = 0.864), and thymoma (THYM; AUC = 0.754), indicating particularly high diagnostic performance in these malignancies (Figure 4).

### 3.3. Prognostic Analysis

**Figure 5:**
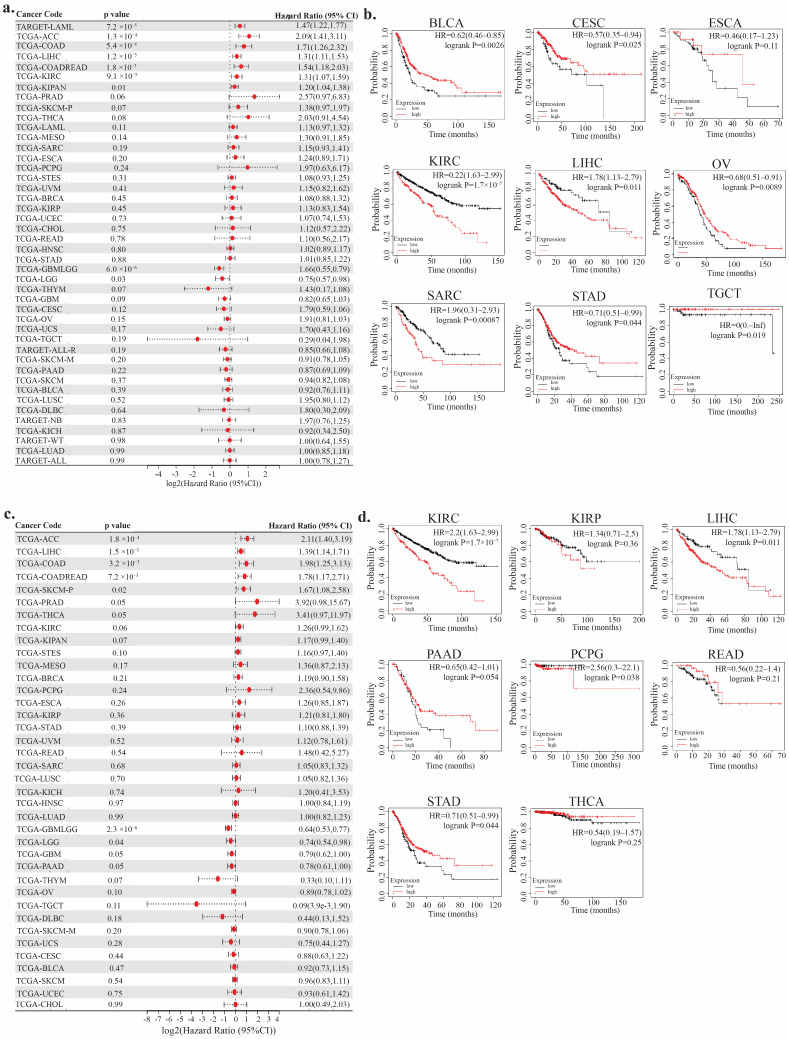
Prognostic value of *TIGD1* expression across cancer types. (**a**) Forest plot of Cox regression analysis for overall survival (OS) in TCGA pan-cancer. (**b**) Kaplan–Meier OS curves for selected tumor types. (**c**) Forest plot of Cox regression analysis for disease-free survival (DFS). (**d**) Kaplan–Meier DFS curves for selected tumor types (*p* < 0.05). CI = confidence interval. In forest plots, lines crossing the null indicate no significance; lines to the right suggest risk, and to the left, protection.

Cox regression analysis identified TIGD1 as a risk factor for poor prognosis in acute myeloid leukemia, the pan-kidney cohort, colon adenocarcinoma, the colorectal cohort, kidney renal clear cell carcinoma, liver hepatocellular carcinoma, and adrenocortical carcinoma, whereas it showed a protective role in the combined glioblastoma and lower-grade glioma cohort and lower-grade glioma (Figure 5a). Kaplan–Meier survival analysis further supported TIGD1 as a risk factor in esophageal carcinoma, LIHC, KIRC, and sarcoma, but indicated a protective role in bladder urothelial carcinoma, cervical squamous cell carcinoma and endocervical adenocarcinoma, ovarian cancer, stomach adenocarcinoma, and testicular germ cell tumors (Figure 5b). Similarly, disease-free survival analysis revealed TIGD1 as a risk factor in ACC, LIHC, COAD, COADREAD, and skin cutaneous melanoma, while a protective association was observed in GBMLGG and LGG (Figure 5c). Kaplan–Meier plots also confirmed a negative prognostic impact in kidney renal papillary cell carcinoma, LIHC, rectum adenocarcinoma, and STAD, whereas favorable prognosis was seen in KIRC, pancreatic adenocarcinoma, pheochromocytoma and paraganglioma, and thyroid carcinoma (Figure 5d).

### 3.4. Immune Correlation of TIGD1 Gene Expression

TE-derived genes have been increasingly recognized for their capacity to modulate immune responses, primarily due to their viral sequence mimicry and ability to engage pattern recognition receptors, such as toll-like receptors and cytosolic nucleic acid sensors [8,9]. These features enable certain TEs to reshape the tumor immune microenvironment and influence responsiveness to immunotherapies. Given TIGD1’s transposable origin, its overexpression in various cancers, and preliminary evidence linking it to immune infiltration, we aimed to explore its immunological relevance across cancer types. Specifically, we analyzed its correlation with immune subtypes, tumor-infiltrating immune cells, immune checkpoint genes, and microenvironmental scores to evaluate its potential role in tumor–immune interactions.

**Figure 6:**
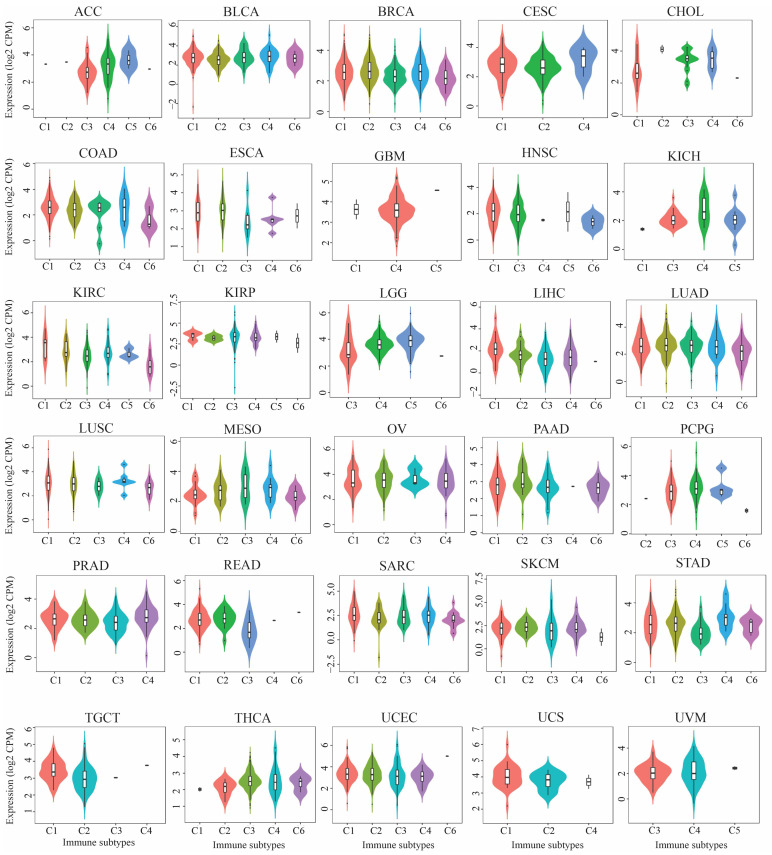
*TIGD1* expression across immune subtypes in pan-cancer. Expression levels (CPM) are shown by immune subtype: C1 (wound healing), C2 (IFN-γ dominant), C3 (inflammatory), C4 (lymphocyte depleted), C5 (immunologically quiet), and C6 (TGF-β dominant). Statistical significance was assessed using the Kruskal–Wallis test (*p* < 0.05). Cancer abbreviations are listed in the “Abbreviation List” section.

**Figure 7:**
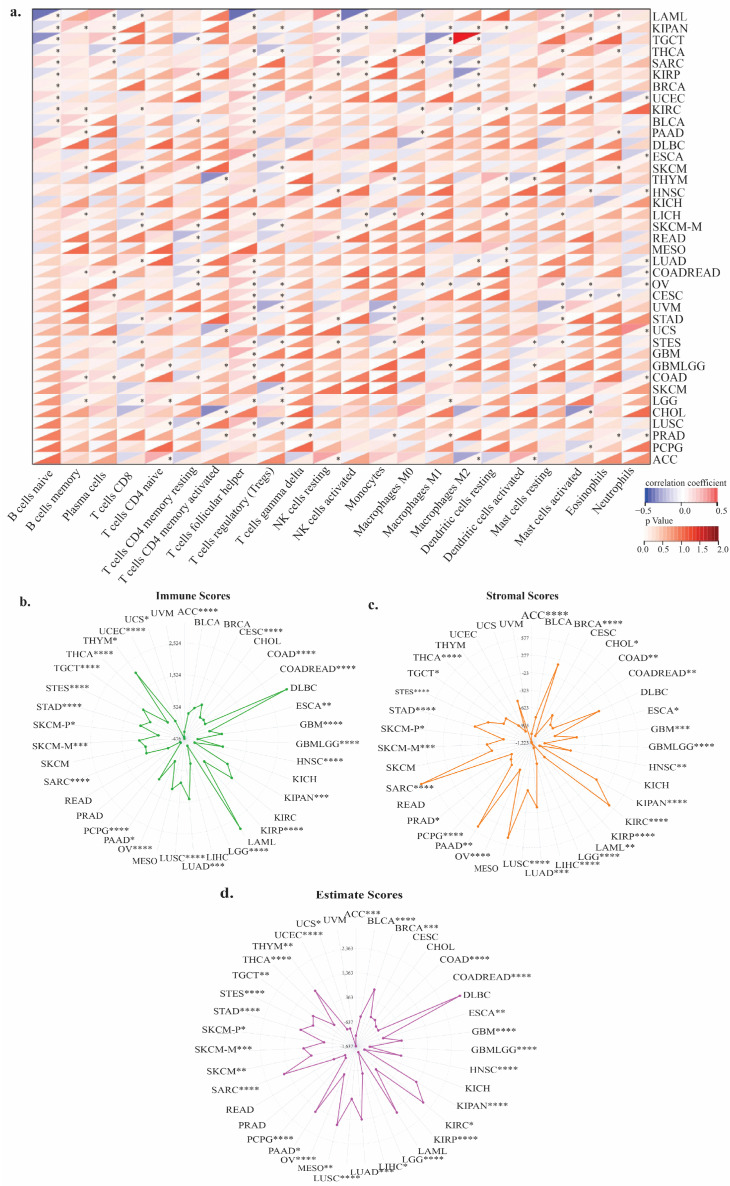
Relationship between *TIGD1* expression and immune-associated features. (**a**) Correlation of *TIGD1* expression with 22 immune cell types based on CIBERSORT analysis; red indicates positive and blue indicates negative correlations. (**b**) Correlation with immune score, (**c**) stromal score, and (**d**) estimate score. Significance: * *p* < 0.05; ** *p* < 0.01; *** *p* < 0.001; **** *p* < 0.0001.

*TIGD1* expression showed significant associations with five immune subtypes across various cancers, as assessed by TISIDB (Figure 6). Further analysis using the CIBERSORT algorithm revealed that *TIGD1* was correlated with immune infiltration in 37 out of 39 cancer types, with notable positive associations with T follicular helper (TFH) cells and negative associations with regulatory T cells (Tregs) (Figure 7a). Additionally, ESTIMATE analysis demonstrated negative correlations between *TIGD1* expression and immune microenvironment scores—stromal scores, immune scores, and estimate scores—in multiple cancers (33 out of 39 cancers), suggesting its role in tumor immunity regulation (Figure 7b–d).

**Figure 8:**
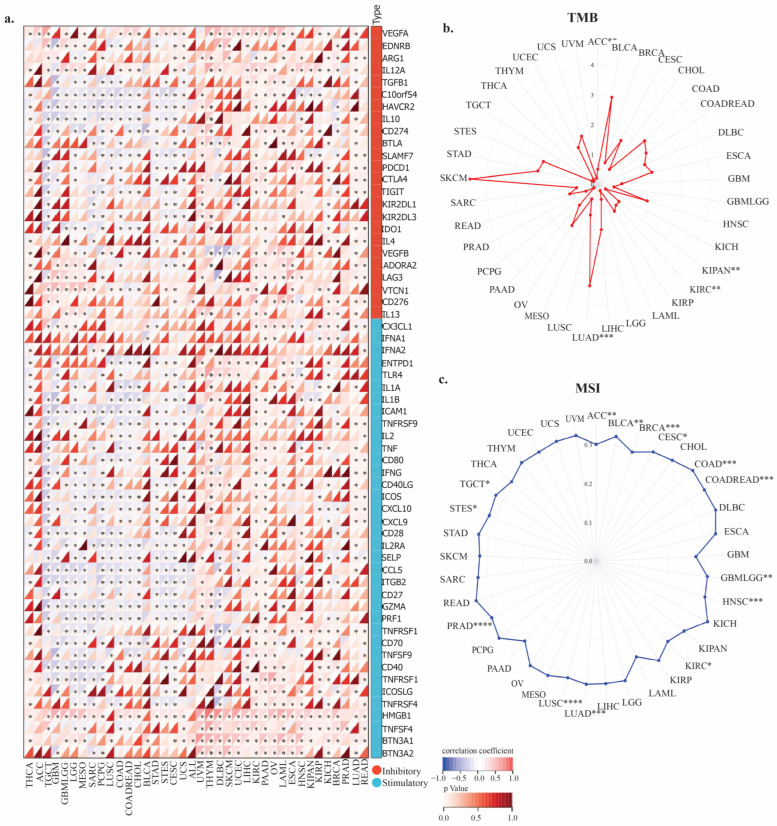
Correlation of *TIGD1* expression with (**a**) immune checkpoint (ICP) genes, (**b**) tumor mutational burden (TMB), and (**c**) microsatellite instability (MSI) across cancers. In (**a**), darker red indicates stronger positive, and darker blue indicates stronger negative correlations. Blue boxes represent stimulatory ICP genes; orange boxes represent inhibitory ones. Significance: * *p* < 0.05; ** *p* < 0.01; *** *p* < 0.001; **** *p* < 0.0001.

Moreover, TIGD1 expression exhibited strong correlations with immune checkpoint-related genes. Significant positive associations were observed in breast invasive carcinoma, kidney renal clear cell carcinoma, liver hepatocellular carcinoma, lung adenocarcinoma, uveal melanoma, thyroid carcinoma, pancreatic adenocarcinoma, and prostate adenocarcinoma. Conversely, negative correlations were noted in testicular germ cell tumors, glioblastoma multiforme, the combined glioblastoma and lower-grade glioma cohort, lower-grade glioma, mesothelioma, lung squamous cell carcinoma, colon adenocarcinoma, colorectal cohort, stomach and esophageal carcinoma, cervical squamous cell carcinoma and endocervical adenocarcinoma, and stomach adenocarcinoma. Particularly noteworthy is the robust positive correlation between TIGD1 and HMGB1 expression across nearly all tumor types, along with significant associations with ICAM1, CCL5, and TNFRSF18 in the majority of cancers (Figure 8a).

Additionally, TIGD1 expression was linked to TMB and MSI, suggesting a potential role in immune evasion and response to immunotherapy (Figure 8b–c). In TMB analysis, positive correlations were identified for LUAD, the pan-kidney cohort (KIPAN), KIRC, and adrenocortical carcinoma (ACC). In MSI analysis, positive correlations were observed in GBMLGG, CESC, LUAD, BRCA, LUSC, TGCT, and bladder urothelial carcinoma (BLCA), whereas negative correlations were identified in COAD, COADREAD, and LUSC.

### 3.5. Gene Set Enrichment Analysis

**Figure 9:**
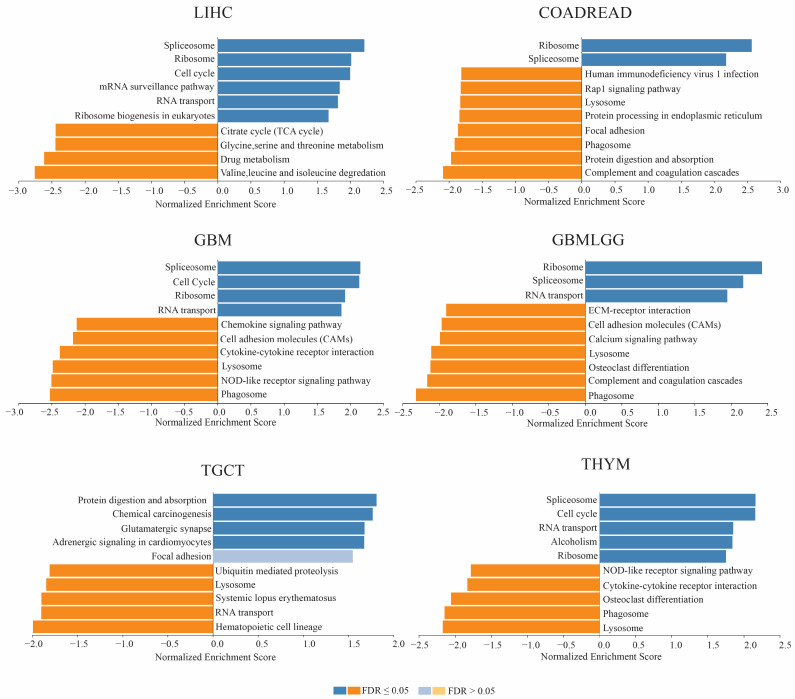
Gene set enrichment analysis (GSEA) of TIGD1 expression in selected cancer types. GSEA was performed to identify biological pathways associated with TIGD1 expression in liver hepatocellular carcinoma (LIHC), colorectal cancer (COADREAD), glioblastoma multiforme (GBM), the combined glioblastoma and lower-grade glioma cohort (GBMLGG), testicular germ cell tumors (TGCT), and thymoma (THYM). The top 10 terms identified by weighted set cover are listed for each cancer. Blue boxes indicate positive correlations, while orange boxes indicate negative correlations. Statistical significance is represented by an FDR ≤ 0.05, highlighted with bright colors.

To elucidate the significance of *TIGD1* in tumorigenesis, we employed GSEA to reveal the functional enrichment of *TIGD1* and KEGG gene sets (Figure 9). The KEGG enrichment items indicated a primary association of *TIGD1* expression with lysosome in 24, spliceosome in 21, and RNA transport in 20 out of 35 TCGA cancers. Obtained pathways can be subcategorized into three. Firstly, *TIGD1* expression displayed a link to RNA processing and translation machinery, including pathways such as RNA transport, spliceosome, ribosome, ribosome biogenesis in eukaryotes, and protein processing in the endoplasmic reticulum. Secondly, *TIGD1* was found associated with cell signaling and communication pathways, including chemokine signaling, cytokine–cytokine receptor interaction, NOD-like receptor signaling, cell cycle, ECM–receptor interaction, and natural killer cell-mediated cytotoxicity. Lastly, *TIGD1* displayed connections to cellular degradation and recycling pathways, encompassing lysosome, phagosome, and endocytosis. Representative GSEA results for cancers providing significant prognostic and diagnostic values are depicted in Figure 8. Comprehensive details of the results are available in the Supplementary Files (Table S2).

### 3.6. Single-Cell Sequencing Analysis

**Figure 10:**
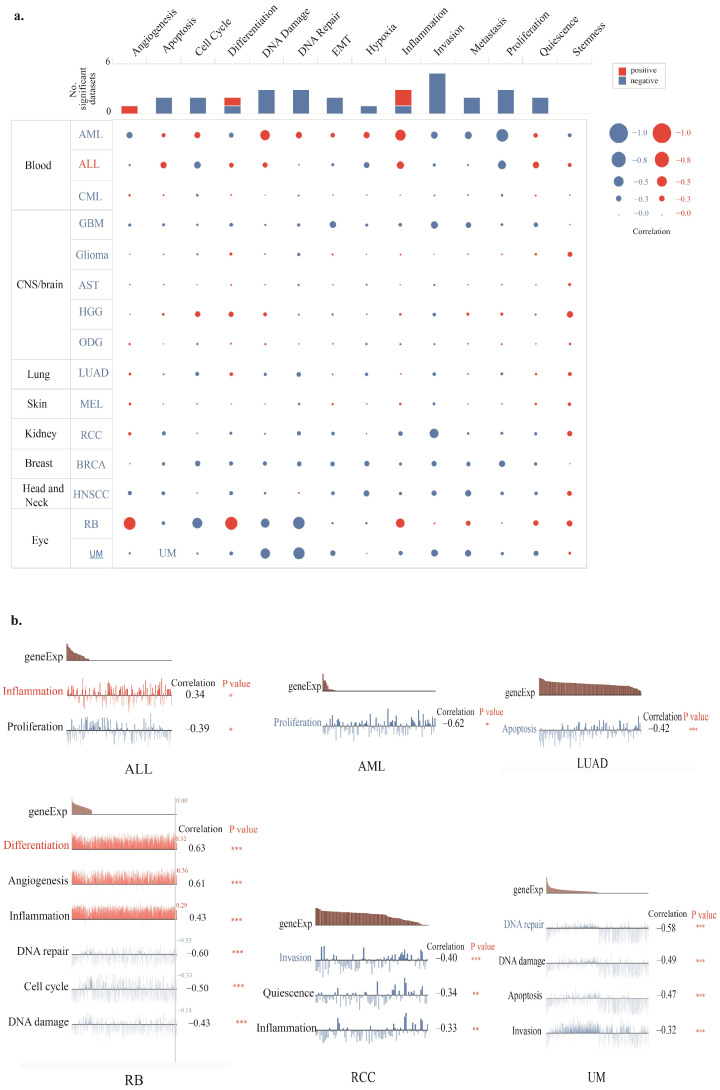
Expression patterns of *TIGD1* in single cells and correlation with the functional condition of the tumor from the CancerSEA database (**a**). A heat map illustrating the relationship between the *TIGD1* expression and functional status of various cancers (**b**). Statistically significant correlations between TIGD1 expression and specific functional states—including proliferation, inflammation, DNA damage, DNA repair, differentiation, and angiogenesis—in acute lymphoblastic leukemia (ALL), acute myeloid leukemia (AML), lung adenocarcinoma (LUAD), retinoblastoma (RB), renal cell carcinoma (RCC), and uveal melanoma (UM) (* *p* < 0.05; ** *p* < 0.01; *** *p* < 0.001).

Single-cell sequencing analysis revealed robust positive correlations between TIGD1 expression and both DNA damage and inflammation in acute myeloid leukemia (AML). In contrast, in retinoblastoma (RB), TIGD1 demonstrated significant positive associations with angiogenesis and cellular differentiation. Additionally, a noteworthy positive correlation between TIGD1 and inflammation was observed in both RB and acute lymphoblastic leukemia (ALL). In RB and uveal melanoma (UM), TIGD1 exhibited marked negative correlations with DNA repair and DNA damage pathways. Notably, cell proliferation showed a strong inverse association with TIGD1 expression in AML (Figure 10a).

As illustrated in Figure 10b, TIGD1 expression was linked to cell proliferation in AML and ALL, inflammation in AML, RB, and renal cell carcinoma (RCC), and DNA repair and damage pathways in RB and UM. Moreover, a strong association with apoptosis was detected in lung adenocarcinoma (LUAD).

Furthermore, the t-SNE plots presented in Figure S1 depict TIGD1 expression patterns in single-cell populations derived from a variety of cancers, including ALL, AML, astrocytoma (AST), breast invasive carcinoma (BRCA), chronic myeloid leukemia (CML), glioblastoma multiforme (GBM), glioma, high-grade glioma (HGG), head and neck squamous cell carcinoma (HNSCC), LUAD, melanoma (MEL), oligodendroglioma (ODG), RB, RCC, and UM.

### 3.7. Correlation Between TIGD1 and Drug Sensitivity

**Figure 11:**
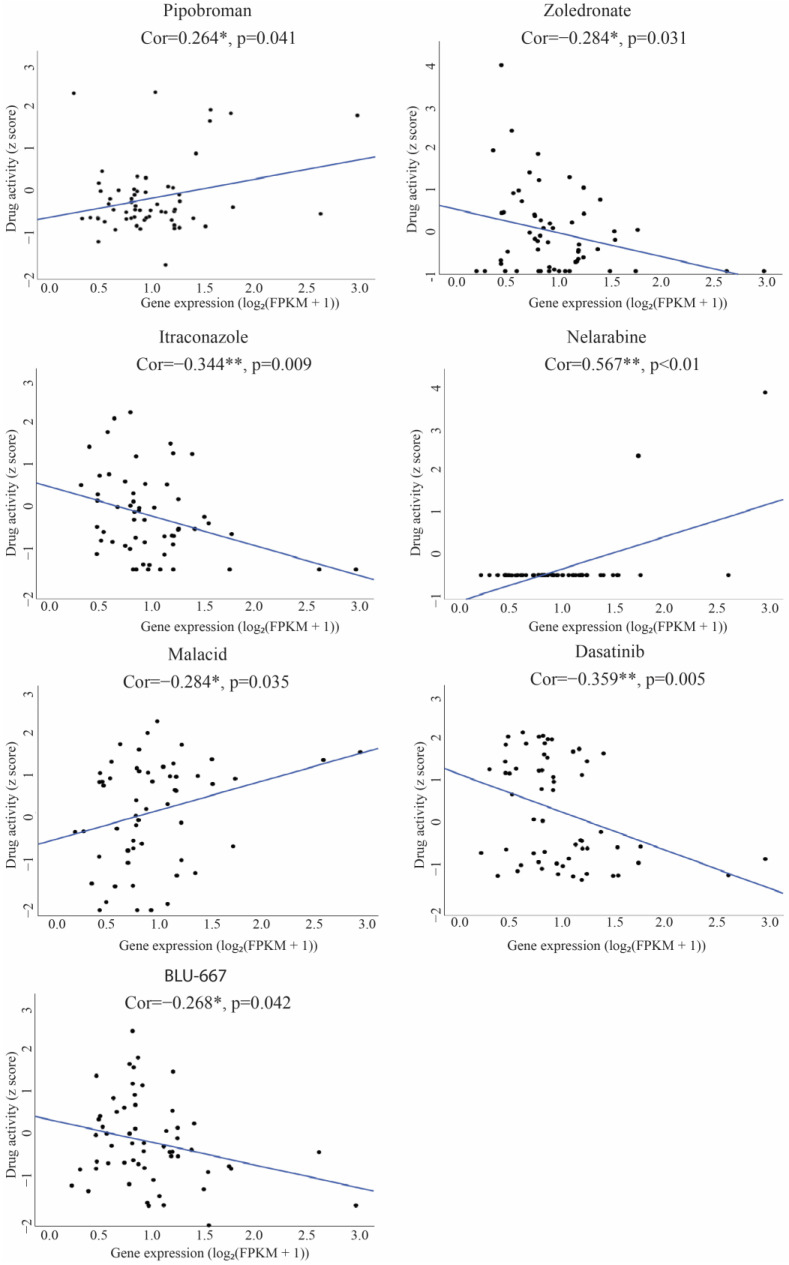
The correlation of *TIGD1* expression and sensitivity of FDA-approved drugs in scatter plots (* *p* < 0.05; ** *p* < 0.01). A positive correlation is indicated by a correlation coefficient (r) > 0, with statistical significance defined by a threshold of *p* < 0.05. The x-axis displays RNA-Seq composite gene expression levels [log_2_(FPKM + 1)], where FPKM denotes Fragments Per Kilobase of Exon per Million reads, and the y-axis represents drug activity level (z-score).

The drug sensitivity correlation with the expression of TIGD1 was obtained using RNA-seq data of the gene and z-scores of more than 300 FDA-approved drugs in the NCI-60 database through the CellMiner platform. TIGD1 expression was positively correlated with sensitivity to Pipobroman and Nelarabine, suggesting a potential role in drug response mechanisms. Conversely, increased TIGD1 expression was negatively associated with the sensitivity of Zoledronate, Itraconazole, Malacid, Dasatinib, and BLU-667, indicating a possible role in chemoresistance (Figure 11). These correlations indicate the possible role of TIGD1 gene expression level in drug resistance mechanisms.

## 4. Discussion

This study provides a comprehensive analysis of *TIGD1* across multiple cancer types, evaluating its expression patterns, prognostic significance, immune interactions, and potential implications in drug resistance. Through an integrative approach, we aimed to elucidate the oncogenic or tumor-suppressive role of *TIGD1* and its functional relevance in tumorigenesis, cancer progression, and therapeutic resistance.

Expression analysis revealed a significant upregulation of the *TIGD1* gene in diverse cancers, including distinct tumor subtypes and pathological stages, implicating its potential involvement in tumorigenesis consistent with the literature [10,11,12,23,24,25]. Interestingly, contrasting downregulation was observed in kidney chromophobe carcinoma, thymoma, and thyroid carcinoma, suggesting a potential dual role of *TIGD1*, acting as an oncogene in certain cancers while exhibiting a tumor-suppressive function in others. This finding underscores the complexity of *TIGD1* in cancer biology and highlights the necessity for context-specific investigations.

Prognosis analyses identified a correlation between elevated *TIGD1* expression and shorter survival in liver hepatocellular carcinoma, colon adenocarcinoma, and the colorectal cancer cohort, reinforcing its pro-tumorigenic role in gastrointestinal cancers. The validation of these findings in colon cancer cell lines further underscores *TIGD1* as a significant prognostic factor and potential therapeutic target in colorectal cancer [23]. In contrast, protective associations were observed in glioma subtypes, pointing toward a role in genomic stability and immune modulation [26]. These divergent roles suggest that *TIGD1* functions in a cancer-type-specific manner, necessitating further mechanistic studies.

ROC analysis provided additional insights into the diagnostic utility of *TIGD1*, demonstrating high predictive accuracy in KICH, TGCT, THCA, and THYM. Collectively, these findings support the potential application of *TIGD1* as a diagnostic and prognostic biomarker across diverse malignancies.

Given that the tumor microenvironment plays a crucial role in cancer progression and response to therapy, we explored *TIGD1*’s immune interactions across cancers. Our findings suggest that *TIGD1* is significantly associated with immune subtypes (C1-C6) and tumor-infiltrating immune cells. Notably, a strong positive correlation with T follicular helper (TFH) cells and a negative correlation with regulatory T (Treg) cells were observed, particularly in tumors with active immune responses. Given that TFH cells are critical for B cell activation and humoral immunity, their correlation with *TIGD1* suggests an immunomodulatory role, potentially influencing tumor progression and immune evasion [27,28]. Furthermore, strong negative correlations with stromal and immune scores in the majority of tumors (excluding lymphoid and rare endocrine cancers) reinforce its association with immune evasion mechanisms.

Further supporting its role in tumor immunity, *TIGD1* expression was significantly correlated with key immune checkpoint genes, including *ICOS, IL10*, and *CTLA-4.* This interplay between *TIGD1* and immune regulators suggests a role in shaping immune surveillance and tumor escape mechanisms. Additionally, high *TIGD1* expression correlated positively with tumor mutation burden in LUAD, KIPAN, and KIRC, as well as microsatellite instability in CESC, LUAD, and BRCA, indicating a potential role for *TIGD1* in immune checkpoint blockade therapy response. These findings suggest that tumors with high *TIGD1* expression may exhibit enhanced sensitivity to immunotherapies, positioning *TIGD1* as a promising immune-related biomarker.

To elucidate the functional role of *TIGD1*, we performed gene set enrichment analysis (GSEA) and single-cell sequencing analysis. GSEA revealed strong associations between *TIGD1* expression and pathways involved in RNA processing (spliceosome, RNA transport), cell signaling (cytokine and chemokine interactions), and cellular degradation (lysosome, endocytosis). Transposable elements, such as *TIGD1*, serve as the main regulators of gene expression via gene editing, through silencing, enhancing, or alternative splicing in human cancers [26,29,30]. Furthermore, the gene expression of *TIGD1* was associated with cell signaling and communication pathways. Disruptions in cellular communication and signaling contribute to carcinogenesis, triggering tumor invasion and metastasis [31,32,33]. Dysregulated cytokine and chemokine signaling impact tumor progression, angiogenesis, and immune modulation [34], suggesting potential connections between *TIGD1*, cancer, and immune response. Lastly, significant associations were found between *TIGD1* expression and cellular degradation and recycling pathways. Those pathways are significant for proteins involved in cellular processes and the expression levels of them, suggesting alterations in cancer metabolism gene expression, which emphasizes the potential role of *TIGD1* expression in tumor progression. Overall, *TIGD1* may play a multifaceted role in promoting carcinogenesis, affecting crucial cellular processes related to gene expression, cellular communication, and homeostasis, with potential interactions between *TIGD1*, cancer, and the immune response.

Single-cell sequencing analysis further demonstrated a strong positive correlation between *TIGD1* expression and genomic instability, inflammation, and angiogenesis, while showing a negative correlation with DNA repair and proliferation. These findings suggest that *TIGD1* may promote carcinogenesis through the dysregulation of cellular stress responses, contributing to increased genomic instability and tumor progression. The observed associations with transposable elements and alternative splicing mechanisms align with previous studies suggesting that *TIGD1* may function as a regulator of gene expression and genome integrity.

Despite advances in cancer treatment, chemotherapy resistance remains a major clinical barrier. Our findings indicate that TIGD1 may be involved in drug response modulation. Specifically, high TIGD1 expression was correlated with increased sensitivity to Pipobroman and Nelarabine—drugs that target DNA synthesis and purine metabolism—suggesting a possible role in DNA damage-related pathways. In contrast, resistance was observed for agents such as Dasatinib and BLU-667 (tyrosine kinase inhibitors), Zoledronate (a bisphosphonate), and Itraconazole (a metabolic modulator with known antiangiogenic activity), implying TIGD1 may contribute to resistance mechanisms involving signaling or metabolic reprogramming. These observations suggest that TIGD1 might influence drug response by modulating pathways such as DNA repair, kinase signaling, or efflux regulation [10,11,23]. While the exact mechanisms remain to be clarified, these associations point to TIGD1 as a potential biomarker for predicting drug response and a candidate for targeting drug resistance in cancer therapy.

Previous studies have linked *TIGD1* to resistance in ovarian and colon cancers [23,35], further supporting its role as a potential molecular target for overcoming therapeutic resistance. The identification of *TIGD1* as a determinant of drug response underscores its significance in precision oncology, warranting further experimental validation to explore its mechanistic involvement in chemotherapy resistance.

To further establish *TIGD1* as a biomarker and therapeutic target, future studies should focus on validating its molecular functions in specific cancer types through experimental and clinical research. Mechanistic studies using in vitro and in vivo models are essential to uncover the precise pathways through which *TIGD1* contributes to tumor progression, immune modulation, and drug resistance. Additionally, integrating multi-omics approaches, including proteomics and metabolomics, may provide deeper insights into the role of *TIGD1* in cancer biology. Given the potential of *TIGD1* as an immune-related biomarker, future clinical investigations should explore its predictive value in immunotherapy response across different malignancies. Further assessment of *TIGD1* expression in patient cohorts receiving immune checkpoint blockade therapy could offer valuable insights into its clinical applicability.

## 5. Conclusions

In conclusion, this study represents the first comprehensive investigation into the intricate association between *TIGD1* and pan-cancer. The observed upregulation of *TIGD1* in multiple malignancies, particularly in colon cancers, suggests a plausible oncogenic role, as evidenced by its correlation with poor patient prognosis. Additionally, *TIGD1* exhibits significant associations with immune cell infiltration, specifically influencing the TFH/Tregs ratio, which may impact tumor immunotherapy efficacy by modulating the TME. Furthermore, *TIGD1* expression is implicated in key cellular processes, including RNA processing, translation, cell signaling, communication, and degradation pathways, highlighting its multifaceted role in carcinogenesis. Notably, this study also establishes a strong link between *TIGD1* expression and drug sensitivity across diverse cancers, reinforcing its potential as a biomarker for precision oncology. As the first study to elucidate the extensive relationship between *TIGD1* and pan-cancer, these findings suggest that *TIGD1* may function as an oncogene and serve as a valuable biomarker in cancer diagnostics, therapeutics, and immunotherapy strategies. Future research should also explore the roles of non-cancer-associated TE-derived genes, which remain largely uncharacterized. Functional experiments assessing their expression and immune relevance may reveal novel biomarker candidates previously overlooked in cancer genomics.

## Data Availability

The study entailed analyzing publicly available datasets, and links to these databases are included in the article. The data that support the findings of this study are available from the corresponding author upon reasonable request.

## Supplementary Materials

The following supporting information can be downloaded at https://www.mdpi.com/article/10.3390/genes16060674/s1, Figure S1: t-SNE plot illustrating *TIGD1* expression in single cells from samples of cancers; Table S1: Diagnostic ROC analysis of *TIGD1* expression across pan-cancers; Table S2: Gene set enrichment analysis (GSEA) of *TIGD1* expression in various TCGA cancers.

## Abbreviations

The following abbreviations are used in this manuscript:

ACC	Adrenocortical carcinoma
AUC	Area under the curve, reflects diagnostic performance from ROC analysis
BLCA	Bladder urothelial carcinoma
BRCA	Breast invasive carcinoma
CESC	Cervical squamous cell carcinoma and endocervical adenocarcinoma
CHOL	Cholangiocarcinoma
COAD	Colon adenocarcinoma
DFS	Disease-free survival
DLBC	Lymphoid Neoplasm Diffuse Large B-cell Lymphoma
ESCA	Esophageal carcinoma
ESTIMATE	Estimation of STromal and Immune cells in MAlignant Tumor tissues using Expression data
FPKM	Fragments Per Kilobase of transcript per Million mapped reads
GBM	Glioblastoma multiforme
GEPIA	Gene Expression Profiling Interactive Analysis, a web tool for RNA-seq expression analysis
GSEA	Gene set enrichment analysis
GTEx	Genotype-Tissue Expression, a resource for studying gene expression across tissues
HR	Hazard ratio
HNSC	Head and neck squamous cell carcinoma
KICH	Kidney chromophobe
KIRC	Kidney renal clear cell carcinoma
KIRP	Kidney renal papillary cell carcinoma
LAML	Acute myeloid leukemia
LGG	Brain lower-grade glioma
LIHC	Liver hepatocellular carcinoma
LUAD	Lung adenocarcinoma
LUSC	Lung squamous cell carcinoma
MESO	Mesothelioma
MSI	Microsatellite instability, a form of genetic hypermutability caused by impaired DNA mismatch repair
OS	Overall survival
OV	Ovarian serous cystadenocarcinoma
PAAD	Pancreatic adenocarcinoma
PCPG	Pheochromocytoma and paraganglioma
PRAD	Prostate adenocarcinoma
READ	Rectum adenocarcinoma
ROC	Receiver operating characteristic, a graphical plot to assess diagnostic accuracy;
SARC	Sarcoma
SKCM	Skin cutaneous melanoma
SPSS	Statistical Package for the Social Sciences
STAD	Stomach adenocarcinoma
TCGA	The Cancer Genome Atlas
TE	Transposable element
TFH	T follicular helper
TGCT	Testicular germ cell tumor
THCA	Thyroid carcinoma
THYM	Thymoma
*TIGD1*	Trigger Transposable Element Derived 1
TISIDB	The Tumor–Immune System Interactions Database
TME	The tumor microenvironment
TMB	Tumor mutational burden, a measure of the number of mutations within a tumor genome
Tregs	T regulatory cells
UCEC	Uterine Corpus Endometrial Carcinoma
UCS	Uterine carcinosarcoma
UVM, UM	Uveal melanoma
WHO	The World Health Organization

## References

[1] Loomans-Kropp H.A., Umar A. (2019). Cancer Prevention and Screening: The next Step in the Era of Precision Medicine. Npj Precis. Oncol..

[2] Zhang Y., Zhang Z. (2020). The History and Advances in Cancer Immunotherapy: Understanding the Characteristics of Tumor-Infiltrating Immune Cells and Their Therapeutic Implications. Cell. Mol. Immunol..

[3] Kazazian H.H., Wong C., Youssoufian H., Scott A.F., Phillips D.G., Antonarakis S.E. (1988). Haemophilia A Resulting from de Novo Insertion of L1 Sequences Represents a Novel Mechanism for Mutation in Man. Nature.

[4] Ogino S., Nosho K., Kirkner G.J., Kawasaki T., Chan A.T., Schernhammer E.S., Giovannucci E.L., Fuchs C.S. (2008). A Cohort Study of Tumoral LINE-1 Hypomethylation and Prognosis in Colon Cancer. J. Natl. Cancer Inst..

[5] Rodić N., Sharma R., Sharma R., Zampella J., Dai L., Taylor M.S., Hruban R.H., Iacobuzio-Donahue C.A., Maitra A., Torbenson M.S. (2014). Long Interspersed Element-1 Protein Expression Is a Hallmark of Many Human Cancers. Am. J. Pathol..

[6] Burns K.H. (2017). Transposable Elements in Cancer. Nat. Rev. Cancer.

[7] Bourque G., Burns K.H., Gehring M., Gorbunova V., Seluanov A., Hammell M., Imbeault M., Izsvák Z., Levin H.L., Macfarlan T.S. (2018). Ten Things You Should Know about Transposable Elements. Genome Biol..

[8] Cubas A.A.d., Dunker W., Zaninovich A., Hongo R.A., Bhatia A., Panda A., Beckermann K.E., Bhanot G., Ganesan S., Karijolich J. (2020). DNA Hypomethylation Promotes Transposable Element Expression and Activation of Immune Signaling in Renal Cell Cancer. JCI Insight.

[9] Wang R., Dong X., Zhang X., Liao J., Cui W., Li W. (2025). Exploring Viral Mimicry Combined with Epigenetics and Tumor Immunity: New Perspectives in Cancer Therapy. Int. J. Biol. Sci..

[10] Zhang G., Feng Z., Zeng Q., Huang P. (2023). Exploring Cancer Dependency Map Genes and Immune Subtypes in Colon Cancer, in Which TIGD1 Contributes to Colon Cancer Progression. Aging.

[11] Wu Z., Lin C., Zhang F., Lu Z., Wang Y., Liu Y., Zhou Z., Li L., Song L. (2023). TIGD1 Function as a Potential Cuproptosis Regulator Following a Novel Cuproptosis-Related Gene Risk Signature in Colorectal Cancer. Cancers.

[12] Yin L., Yan J., Wang Y., Sun Q. (2019). TIGD1, a Gene of Unknown Function, Involves Cell-cycle Progression and Correlates with Poor Prognosis in Human Cancer. J. Cell. Biochem..

[13] Xia L., Yang Z., Xv M., Wang G., Mao Y., Yang Y., Tang J. (2024). Bioinformatics Analysis and Experimental Verification of TIGD1 in Non-Small Cell Lung Cancer. Front. Med..

[14] Li T., Fu J., Zeng Z., Cohen D., Li J., Chen Q., Li B., Liu X.S. (2020). TIMER2.0 for Analysis of Tumor-Infiltrating Immune Cells. Nucleic Acids Res..

[15] Tang Z., Kang B., Li C., Chen T., Zhang Z. (2019). GEPIA2: An Enhanced Web Server for Large-Scale Expression Profiling and Interactive Analysis. Nucleic Acids Res..

[16] Ru B., Wong C.N., Tong Y., Zhong J.Y., Zhong S.S.W., Wu W.C., Chu K.C., Wong C.Y., Lau C.Y., Chen I. (2019). TISIDB: An Integrated Repository Portal for Tumor–Immune System Interactions. Bioinformatics.

[17] Goldman M.J., Craft B., Hastie M., Repečka K., McDade F., Kamath A., Banerjee A., Luo Y., Rogers D., Brooks A.N. (2020). Visualizing and Interpreting Cancer Genomics Data via the Xena Platform. Nat. Biotechnol..

[18] Janssens A.C.J.W., Martens F.K. (2020). Reflection on Modern Methods: Revisiting the Area under the ROC Curve. Int. J. Epidemiol..

[19] Nagy Á., Munkácsy G., Győrffy B. (2021). Pancancer Survival Analysis of Cancer Hallmark Genes. Sci. Rep..

[20] Vasaikar S.V., Straub P., Wang J., Zhang B. (2017). LinkedOmics: Analyzing Multi-Omics Data within and across 32 Cancer Types. Nucleic Acids Res..

[21] Yuan H., Yan M., Zhang G., Liu W., Deng C., Liao G., Xu L., Luo T., Yan H., Long Z. (2019). CancerSEA: A Cancer Single-Cell State Atlas. Nucleic Acids Res..

[22] Pommier Y., Reinhold W.C., Sunshine M., Varma S.H., Kohn K.W., Doroshow J.H. (2012). 268 CellMiner: A Web-Based Suite of Genomic and Pharmacologic Tools to Explore Transcript and Drug Patterns in the NCI-60 Cell Line Set. Eur. J. Cancer.

[23] Zou J., Zhang H., Wu Z., Hu W., Zhang T., Xie H., Huang Y., Zhou H. (2024). TIGD1 Is an Independent Prognostic Factor That Promotes the Progression of Colon Cancer. Cancer Biother. Radiopharm..

[24] Qiao X., Zhu L., Song R., Shang C., Guo Y. (2023). A Novel Oncogene Trigger Transposable Element Derived-1 Promotes Oral Squamous Cell Carcinoma Progression via Evoking Immune Inhibition. Mol. Carcinog..

[25] Ge X., Liu Z., Jiao X., Yin X., Wang X., Li G. (2021). Establishment and Validation of a Gene Signature-Based Prognostic Model to Improve Survival Prediction in Adrenocortical Carcinoma Patients. Int. J. Endocrinol..

[26] Gebrie A. (2023). Transposable Elements as Essential Elements in the Control of Gene Expression. Mob. DNA.

[27] Guo Z., Liang H., Xu Y., Liu L., Ren X., Zhang S., Wei S., Xu P. (2017). The Role of Circulating T Follicular Helper Cells and Regulatory Cells in Non-Small Cell Lung Cancer Patients. Scand. J. Immunol..

[28] Hetta H.F., Elkady A., Yahia R., Meshall A.K., Saad M.M., Mekky M.A., Al-Kadmy I.M.S. (2020). T Follicular Helper and T Follicular Regulatory Cells in Colorectal Cancer: A Complex Interplay. J. Immunol. Methods.

[29] Anwar S.L., Wulaningsih W., Lehmann U. (2017). Transposable Elements in Human Cancer: Causes and Consequences of Deregulation. Int. J. Mol. Sci..

[30] Yanar S., Albayrak M.G.B., Kasap M., Akpinar G. (2024). From Androgen Dependence to Independence in Prostate Cancer: Unraveling Therapeutic Potential and Proteomic Landscape of Hydroxychloroquine as an Autophagy Inhibitor. OMICS J. Integr. Biol..

[31] You M., Xie Z., Zhang N., Zhang Y., Xiao D., Liu S., Zhuang W., Li L., Tao Y. (2023). Signaling Pathways in Cancer Metabolism: Mechanisms and Therapeutic Targets. Signal Transduct. Target. Ther..

[32] Hanahan D., Weinberg R.A. (2011). Hallmarks of Cancer: The Next Generation. Cell.

[33] Perry D.K., Kolesnick R.N. (2004). Signal Transduction in Cancer. Cancer Treat. Res..

[34] Bule P., Aguiar S.I., Aires-Da-Silva F., Dias J.N.R. (2021). Chemokine-Directed Tumor Microenvironment Modulation in Cancer Immunotherapy. Int. J. Mol. Sci..

[35] Mucaki E.J., Zhao J.Z.L., Lizotte D.J., Rogan P.K. (2019). Predicting Responses to Platin Chemotherapy Agents with Biochemically-Inspired Machine Learning. Signal Transduct. Target. Ther..

