# Supplementary material for: Beyond Transposons: TIGD1 as a Pan-Cancer Biomarker and Immune Modulator
Source: Genes (Basel). 2025 May 30;16(6):674. doi: 10.3390/genes16060674 (PMC12192532; doi:10.3390/genes16060674)
Supplement: Supplementary file 1 [file genes-16-00674-s001.zip › Table S1.pdf]

Table S1: Diagnostic ROC Analysis of TIGD1 Expression Across Pan-Cancers. This table presents the Area Under the Curve (AUC) values with 95% Confidence Intervals (CI), cutoff values, p-values, sensitivity, specificity, and overall diagnostic value for each cancer type. AUC values were interpreted as follows: 0.5–0.6 indicates "no diagnostic value," 0.6–0.75 indicates "moderate diagnostic value," and 0.75–1.0 indicates "high diagnostic value." Cancer type abbreviations are provided in the "Abbreviation List" section.

Cancer Type	AUC (95% CI)	Cutoff	p-value	Sensitivity	Specificity	Diagnostic Value
**ACC**	0.551 (0.491-0.610)	>7.182	0.1831	63.29	53.43	Non
**BLCA**	0.647 (0.615-0.679)	≤7.47	<0.0001	74.47	54.02	Moderate
**BRCA**	0.661 (0.643-0.680	≤7.02	<0.0001	63.87	64.13	Moderate
**CESC**	0.651 (0.612-0.688	≤7.25	<0.0001	65.05	63.75	Moderate
**CHOL**	0.632 (0.524-0.732	≤8.49	0.0263	84.44	44.44	Moderate
**COAD**	0.640 (0.611-0.668)	≤7.06	<0.0001	62.40	62.83	Moderate
**DLBC**	0.666 (0.562-0.759)	≤7.55	0.0042	81.25	59.57	Moderate
**ESCA**	0.534 (0.503-0.565)	>5.869	0.1185	98.98	10.19	Non
**GBM**	0.687 (0.667-0.708)	≤8.03	<0.0001	67.47	69.13	Moderate
**HNSC**	0.618 (0.589-0.647)	≤6.8	<0.0001	65.19	56.03	Moderate
**KICH**	0.770 (0.746-0.793	≤7.04	<0.0001	73.63	71.48	High
**KIRC**	0.727 (0.706-0.747	≤7.5	<0.0001	83.66	55.54	Moderate
**KIRP**	0.557 (0.532-0.582)	≤8.42	0.0005	89.78	26.01	Non
**LAML**	0.528 (0.463-0.592)	≤8.22	0.4662	52.60	74.29	Non
**LGG**	0.618(0.598-0.638)	≤8.1	<0.0001	55.58	64.75	Moderate
**LIHC**	0.595 (0.563-0.627)	≤6.84	<0.0001	75.18	45.57	Non
**LUAD**	0.669 (0.648-0.690)	≤7.25	<0.0001	71.01	60.40	Moderate
**LUSC**	0.549 (0.527-0.572)	≤7.58	0.0004	64.13	46.20	Non
**OV**	0.625 (0.591-0.658)	≤8.55	<0.0001	77.92	45.53	Moderate
**PAAD**	0.610(0.567-0.651)	≤7.46	<0.0001	73.22	51.72	Moderate
**PCPG**	0.540 (0.489-0.590	>7.178	0.1709	59.89	52.94	Non
**PRAD**	0.718 (0.691-0.743)	≤7.22	<0.0001	75.09	63.12	Moderate
**READ**	0.575(0.540-0.609)	≤7.15	0.0018	58.82	58.90	Non
**SARC**	0.632 (0.589-0.673)	≤7.48	<0.0001	77.36	44.70	Moderate
**SKCM**	0.674 (0.651-0.696)	≤7.02	<0.0001	65.12	67.16	Moderate
**STAD**	0.666 (0.637-0.695)	≤7.48	<0.0001	77.56	55.14	Moderate
**TGCT**	0.783 (0.742-0.820)	≤8.27	<0.0001	91.37	62.38	High
**THCA**	0.864 (0.839-0.887)	≤7.67	<0.0001	93.88	82.73	High
**THYM**	0.754 (0.695-0.807)	≤8.74	<0.0001	88.52	66.94	High
**UCEC**	0.587 (0.549-0.624)	≤8.09	0.0025	66.67	49.63	Non
**UCS**	0.586 (0.512-0.656)	>7.541	0.0396	92.98	30.37	Non
**UVM**	0.620 (0.540-0.696)	≤6.54	0.0076	50.00	72.15	Moderate
