# Supplementary material for: Beyond Transposons: TIGD1 as a Pan-Cancer Biomarker and Immune Modulator
Source: Genes (Basel). 2025 May 30;16(6):674. doi: 10.3390/genes16060674 (PMC12192532; doi:10.3390/genes16060674)
Supplement: Supplementary file 1 [file genes-16-00674-s001.zip › Table S2.pdf]

Table S2: Gene Set Enrichment Analysis (GSEA) of TIGD1 expression in various TCGA cancers. The table provides enrichment scores (ES), normalized enrichment scores (NES), p-values, and false discovery rates (FDR) for the top pathways associated with TIGD1 expression across different cancer types. The leading-edge number reflects the number of genes in the pathway that drive the enrichment signal. Pathway descriptions and sizes are also included. Pathways are considered statistically significant at FDR ≤ 0.05.

TCGA Cancers	Gene Set	Description	Size	Leading Edge Number	ES	NES	P Value	FDR
SKCM	hsa05221	Acute myeloid leukemia	66	28	-0.45056	-16.890	<2.2e-16	0.055871
KIRC	hsa04520	Adherens junction	66	33	-0.60169	-20.310	<2.2e-16	0.0058037
TGCT	hsa04261	Adrenergic signaling in cardiomyocytes	144	55	0.43706	16.790	<2.2e-16	0.023376
ACC	hsa05034	Alcoholism	169	61	0.43760	17.846	<2.2e-16	0.0098621
BLCA	hsa05034	Alcoholism	174	55	0.51115	18.083	<2.2e-16	0.0078358
BRCA	hsa05034	Alcoholism	174	58	0.44636	14.646	<2.2e-16	0.11159
CESC	hsa05034	Alcoholism	173	57	0.48760	15.652	<2.2e-16	0.89872
LUSC	hsa05034	Alcoholism	174	69	0.48622	17.704	<2.2e-16	0.0044308
THYM	hsa05034	Alcoholism	171	82	0.53339	18.410	<2.2e-16	0.0023510
UCEC	hsa00520	Amino sugar and nucleotide sugar metabolism	48	32	-0.66631	-23.524	<2.2e-16	<2.2e-16
PRAD	hsa05146	Amoebiasis	93	51	-0.51326	-18.003	<2.2e-16	0.016274
UVM	hsa04152	AMPK signaling pathway	120	18	-0.26912	-11.531	<2.2e-16	0.57352
CESC	hsa04210	Apoptosis	133	51	-0.53212	-21.764	<2.2e-16	0.00046924
UCS	hsa04210	Apoptosis	132	40	-0.44452	-21.420	<2.2e-16	<2.2e-16

KICH	hsa04360	Axon guidance	173	54	-0.40787	-15.928	<2.2e-16	0.11710
KIRC	hsa04360	Axon guidance	173	53	-0.46333	-18.128	<2.2e-16	0.031340
LUAD	hsa04360	Axon guidance	173	78	-0.55360	-21.584	<2.2e-16	<2.2e-16
PRAD	hsa04360	Axon guidance	173	68	-0.46575	-17.855	<2.2e-16	0.019583
UVM	hsa01230	Biosynthesis of amino acids	69	19	-0.41958	-15.145	<2.2e-16	0.38292
SKCM	hsa05219	Bladder cancer	40	18	-0.47425	-16.982	<2.2e-16	0.056098
LUSC	hsa04625	C-type lectin receptor signaling pathway	104	59	-0.59484	-20.615	<2.2e-16	<2.2e-16
LGG	hsa04020	Calcium signaling pathway	181	78	-0.52796	-19.936	<2.2e-16	0.0019068
KIRP	hsa01200	Carbon metabolism	110	55	-0.71690	-26.010	<2.2e-16	<2.2e-16
PCPG	hsa01200	Carbon metabolism	110	35	-0.53441	-19.534	<2.2e-16	0.0010411
SKCM	hsa01200	Carbon metabolism	110	34	-0.45270	-17.976	<2.2e-16	0.030899
UCEC	hsa01200	Carbon metabolism	110	42	-0.52622	-21.874	<2.2e-16	<2.2e-16
ACC	hsa04514	Cell adhesion molecules (CAMs)	135	48	-0.51844	-19.514	<2.2e-16	0.00075645
BLCA	hsa04514	Cell adhesion molecules (CAMs)	137	72	-0.67747	-26.035	<2.2e-16	<2.2e-16
LGG	hsa04514	Cell adhesion molecules (CAMs)	137	67	-0.48121	-17.667	<2.2e-16	0.0095341

GBM	hsa04514	Cell adhesion molecules (CAMs)	136	61	-0.56992	-21.775	<2.2e-16	<2.2e-16
LUAD	hsa04514	Cell adhesion molecules (CAMs)	137	67	-0.59937	-22.941	<2.2e-16	<2.2e-16
LUSC	hsa04514	Cell adhesion molecules (CAMs)	137	81	-0.65881	-23.503	<2.2e-16	<2.2e-16
MESO	hsa04514	Cell adhesion molecules (CAMs)	135	61	-0.53681	-22.933	<2.2e-16	<2.2e-16
OV	hsa04514	Cell adhesion molecules (CAMs)	137	71	-0.65005	-23.941	<2.2e-16	<2.2e-16
PAAD	hsa04514	Cell adhesion molecules (CAMs)	136	57	-0.56032	-21.513	<2.2e-16	<2.2e-16
STAD	hsa04514	Cell adhesion molecules (CAMs)	137	71	-0.69028	-27.039	<2.2e-16	<2.2e-16
STES	hsa04514	Cell adhesion molecules (CAMs)	137	63	-0.68072	-28.222	<2.2e-16	<2.2e-16
THCA	hsa04514	Cell adhesion molecules (CAMs)	136	67	-0.59571	-22.416	<2.2e-16	<2.2e-16
ACC	hsa04110	Cell cycle	118	44	0.40364	15.504	<2.2e-16	0.057812
BRCA	hsa04110	Cell cycle	118	47	0.60375	19.063	<2.2e-16	0.00020524
GBM	hsa04110	Cell cycle	118	64	0.65292	21.351	<2.2e-16	<2.2e-16
LIHC	hsa04110	Cell cycle	118	39	0.65043	19.975	<2.2e-16	<2.2e-16
THYM	hsa04110	Cell cycle	118	53	0.64837	21.571	<2.2e-16	<2.2e-16
KIPAN	hsa04218	Cellular senescence	155	58	-0.47935	-17.701	<2.2e-16	0.022024
KICH	hsa04022	cGMP-PKG signaling pathway	158	40	-0.40378	-15.406	<2.2e-16	0.12581

KIPAN	hsa04022	cGMP-PKG signaling pathway	160	68	-0.54324	-20.081	<2.2e-16	<2.2e-16
STAD	hsa04022	cGMP-PKG signaling pathway	160	57	-0.51401	-20.897	<2.2e-16	<2.2e-16
TGCT	hsa05204	Chemical carcinogenesis	75	40	0.50676	17.729	<2.2e-16	0.023353
ACC	hsa04062	Chemokine signaling pathway	185	93	-0.54291	-20.464	<2.2e-16	0.00034040
LAML	hsa04062	Chemokine signaling pathway	184	55	-0.58016	-21.637	<2.2e-16	<2.2e-16
GBM	hsa04062	Chemokine signaling pathway	185	87	-0.53951	-21.216	<2.2e-16	<2.2e-16
HNSC	hsa04062	Chemokine signaling pathway	185	77	-0.52555	-23.868	<2.2e-16	<2.2e-16
LUAD	hsa04062	Chemokine signaling pathway	185	73	-0.55610	-22.684	<2.2e-16	<2.2e-16
DLBC	hsa04062	Chemokine signaling pathway	185	77	-0.43872	-19.932	<2.2e-16	0.00047499
MESO	hsa04062	Chemokine signaling pathway	185	88	-0.49152	-21.573	<2.2e-16	<2.2e-16
OV	hsa04062	Chemokine signaling pathway	185	92	-0.67874	-25.582	<2.2e-16	<2.2e-16
PAAD	hsa04062	Chemokine signaling pathway	185	76	-0.48995	-19.238	<2.2e-16	0.00075320
SARC	hsa04062	Chemokine signaling pathway	185	91	-0.56821	-23.452	<2.2e-16	<2.2e-16
THCA	hsa04062	Chemokine signaling pathway	185	73	-0.53651	-20.429	<2.2e-16	<2.2e-16
KIRC	hsa00020	Citrate cycle (TCA cycle)	30	16	-0.69557	-19.970	<2.2e-16	0.0087056

LIHC	hsa00020	Citrate cycle (TCA cycle)	30	21	-0.80566	-24.369	<2.2e-16	<2.2e-16
KIPAN	hsa00020	Citrate cycle (TCA cycle)	30	16	-0.61694	-17.138	<2.2e-16	0.023433
COADREAD	hsa04610	Complement and coagulation cascades	78	36	-0.58995	-20.876	<2.2e-16	<2.2e-16
SARC	hsa04610	Complement and coagulation cascades	78	36	-0.63999	-24.557	<2.2e-16	<2.2e-16
CHOL	hsa04610	Complement and coagulation cascades	78	36	-0.58995	-20.826	<2.2e-16	0.00079252
BLCA	hsa04060	Cytokine-cytokine receptor interaction	280	135	-0.61270	-25.869	<2.2e-16	<2.2e-16
CESC	hsa04060	Cytokine-cytokine receptor interaction	277	92	-0.46020	-20.028	<2.2e-16	0.00082807
GBM	hsa04060	Cytokine-cytokine receptor interaction	274	111	-0.56787	-23.761	<2.2e-16	<2.2e-16
LUSC	hsa04060	Cytokine-cytokine receptor interaction	281	142	-0.63621	-24.837	<2.2e-16	<2.2e-16
MESO	hsa04060	Cytokine-cytokine receptor interaction	271	118	-0.50757	-22.608	<2.2e-16	<2.2e-16
OV	hsa04060	Cytokine-cytokine receptor interaction	278	122	-0.60241	-23.346	<2.2e-16	<2.2e-16
SARC	hsa04060	Cytokine-cytokine receptor interaction	279	84	-0.46625	-22.097	<2.2e-16	<2.2e-16
STAD	hsa04060	Cytokine-cytokine receptor interaction	281	107	-0.58605	-25.505	<2.2e-16	<2.2e-16
STES	hsa04060	Cytokine-cytokine receptor interaction	281	106	-0.58147	-24.180	<2.2e-16	<2.2e-16
THYM	hsa04060	Cytokine-cytokine receptor interaction	275	103	-0.43230	-18.273	<2.2e-16	0.0049865

	Gene Set	Description	Size	Leading Edge Number	ES	NES	P Value	**FDR**
LIHC	hsa00982	Drug metabolism	66	36	-0.75418	-26.140	<2.2e-16	<2.2e-16
LGG	hsa04512	ECM-receptor interaction	80	44	-0.52173	-17.647	<2.2e-16	0.0093334
ESCA	hsa04512	ECM-receptor interaction	80	27	-0.46628	-19.484	<2.2e-16	0.0056356
GBMLGG	hsa04512	ECM-receptor interaction	80	27	-0.46628	-19.484	<2.2e-16	0.0056356
KICH	hsa04512	ECM-receptor interaction	80	34	-0.53006	-18.426	<2.2e-16	0.039259
KIRC	hsa04512	ECM-receptor interaction	80	37	-0.52466	-18.476	<2.2e-16	0.028293
LAML	hsa04144	Endocytosis	227	83	-0.57950	-22.342	<2.2e-16	<2.2e-16
BRCA	hsa04144	Endocytosis	228	58	-0.48997	-18.649	<2.2e-16	0.0046961
CESC	hsa04144	Endocytosis	228	86	-0.52393	-22.364	<2.2e-16	<2.2e-16
HNSC	hsa04144	Endocytosis	228	84	-0.51804	-23.947	<2.2e-16	<2.2e-16
LUSC	hsa04144	Endocytosis	228	68	-0.52864	-20.377	<2.2e-16	<2.2e-16
OV	hsa04144	Endocytosis	228	93	-0.55305	-21.660	<2.2e-16	<2.2e-16
THCA	hsa04144	Endocytosis	228	91	-0.60052	-23.712	<2.2e-16	<2.2e-16

CESC	hsa05169	Epstein-Barr virus infection	193	85	-0.48987	-21.339	<2.2e-16	0.00082807
MESO	hsa05169	Epstein-Barr virus infection	189	78	-0.48247	-21.610	<2.2e-16	<2.2e-16
THCA	hsa05169	Epstein-Barr virus infection	197	75	-0.57386	-22.086	<2.2e-16	<2.2e-16
KIRC	hsa04216	Ferroptosis	40	17	-0.61114	-19.534	<2.2e-16	0.0087056
KIRP	hsa04216	Ferroptosis	39	20	-0.67498	-20.683	<2.2e-16	<2.2e-16
BRCA	hsa04510	Focal adhesion	194	76	-0.50686	-18.697	<2.2e-16	0.0048303
COADREAD	hsa04510	Focal adhesion	194	79	-0.47125	-18.651	<2.2e-16	0.0040043
HNSC	hsa04510	Focal adhesion	194	76	-0.60489	-27.473	<2.2e-16	<2.2e-16
LUAD	hsa04510	Focal adhesion	194	96	-0.65029	-25.287	<2.2e-16	<2.2e-16
KIPAN	hsa04510	Focal adhesion	194	89	-0.50390	-18.134	<2.2e-16	0.014132
PAAD	hsa04510	Focal adhesion	194	90	-0.49275	-19.806	<2.2e-16	0.00088838
PCPG	hsa04510	Focal adhesion	194	68	-0.57044	-22.020	<2.2e-16	<2.2e-16
STES	hsa04510	Focal adhesion	194	80	-0.60883	-24.567	<2.2e-16	<2.2e-16
TGCT	hsa04510	Focal adhesion	194	73	0.39107	15.495	<2.2e-16	0.068933
CHOL	hsa04510	Focal adhesion	194	79	-0.47125	-18.827	<2.2e-16	0.0027474

KICH	hsa04068	FoxO signaling pathway	127	41	-0.40891	-15.547	<2.2e-16	0.11840
KIRC	hsa04068	FoxO signaling pathway	129	44	-0.47054	-17.944	<2.2e-16	0.044319
KIPAN	hsa04068	FoxO signaling pathway	129	57	-0.55383	-19.903	<2.2e-16	<2.2e-16
UVM	hsa00051	Fructose and mannose metabolism	31	10	-0.61317	-20.442	<2.2e-16	0.021348
SKCM	hsa00052	Galactose metabolism	29	18	-0.69245	-22.303	<2.2e-16	<2.2e-16
TGCT	hsa04724	Glutamatergic synapse	111	48	0.46216	16.831	<2.2e-16	0.023718
LIHC	hsa00260	Glycine, serine and threonine metabolism	39	27	-0.81180	-24.399	<2.2e-16	<2.2e-16
KIRP	hsa00010	Glycolysis / Gluconeogenesis	66	32	-0.60689	-20.743	<2.2e-16	<2.2e-16
SKCM	hsa00534	Glycosaminoglycan biosynthesis	24	10	-0.56903	-17.480	<2.2e-16	0.039509
KIPAN	hsa05332	Graft-versus-host disease	37	26	-0.59191	-17.286	<2.2e-16	0.023125
ACC	hsa04640	Hematopoietic cell lineage	92	50	-0.62826	-22.086	<2.2e-16	<2.2e-16
PCPG	hsa04640	Hematopoietic cell lineage	93	44	-0.57085	-20.095	<2.2e-16	0.00058993
TGCT	hsa04640	Hematopoietic cell lineage	93	47	-0.51896	-19.917	<2.2e-16	0.00097635
UCS	hsa04640	Hematopoietic cell lineage	92	47	-0.47963	-21.089	<2.2e-16	<2.2e-16
KIRP	hsa03440	Homologous recombination	34	15	0.72432	17.247	<2.2e-16	0.013387
UVM	hsa03440	Homologous recombination	34	17	0.66147	16.217	<2.2e-16	0.11415

COADREAD	hsa05170	Human immunodeficiency virus 1 infection	196	54	-0.45026	-18.150	<2.2e-16	0.0046129
CHOL	hsa05170	Human immunodeficiency virus 1 infection	196	54	-0.45026	-18.183	<2.2e-16	0.0054155
HNSC	hsa05016	Huntington disease	174	72	0.48906	14.763	<2.2e-16	0.072070
MESO	hsa05016	Huntington disease	174	60	0.41420	15.355	<2.2e-16	0.12920
PRAD	hsa05016	Huntington disease	174	70	0.53584	14.780	<2.2e-16	0.12756
UCEC	hsa04657	IL-17 signaling pathway	89	42	-0.50651	-20.736	<2.2e-16	0.00046989
LAML	hsa05164	Influenza A	160	51	-0.56505	-21.230	<2.2e-16	0.00037147
UVM	hsa04910	Insulin signaling pathway	137	25	-0.27423	-11.747	<2.2e-16	0.54300
HNSC	hsa04630	JAK-STAT signaling pathway	158	76	-0.57832	-24.960	<2.2e-16	<2.2e-16
PAAD	hsa04630	JAK-STAT signaling pathway	153	56	-0.48084	-18.850	<2.2e-16	0.0016169
PRAD	hsa04630	JAK-STAT signaling pathway	156	45	-0.48228	-18.051	<2.2e-16	0.017583
LAML	hsa05167	Kaposi sarcoma-associated herpesvirus infection	179	63	-0.52293	-20.363	<2.2e-16	0.00025841
ESCA	hsa04670	Leukocyte transendothelial migration	109	47	-0.46248	-20.253	<2.2e-16	0.0044402
GBMLGG	hsa04670	Leukocyte transendothelial migration	109	47	-0.46248	-20.253	<2.2e-16	0.0044402
KIRC	hsa04670	Leukocyte transendothelial migration	109	44	-0.43504	-17.084	<2.2e-16	0.053150
ACC	hsa04142	Lysosome	121	57	-0.53343	-19.438	<2.2e-16	0.00071663

BLCA	hsa04142	Lysosome	121	59	-0.63902	-24.392	<2.2e-16	<2.2e-16
LAML	hsa04142	Lysosome	121	57	-0.68334	-24.680	<2.2e-16	<2.2e-16
LGG	hsa04142	Lysosome	121	65	-0.50840	-19.153	<2.2e-16	0.0024516
BRCA	hsa04142	Lysosome	121	54	-0.56343	-19.804	<2.2e-16	<2.2e-16
COADRE AD	hsa04142	Lysosome	121	41	-0.49509	-18.308	<2.2e-16	0.0042227
GBM	hsa04142	Lysosome	121	69	-0.67086	-24.836	<2.2e-16	<2.2e-16
KIRP	hsa04142	Lysosome	121	63	-0.65017	-24.290	<2.2e-16	<2.2e-16
LUAD	hsa04142	Lysosome	121	50	-0.57327	-21.135	<2.2e-16	<2.2e-16
LUSC	hsa04142	Lysosome	121	62	-0.67583	-23.906	<2.2e-16	<2.2e-16
DLBC	hsa04142	Lysosome	121	59	-0.66528	-28.347	<2.2e-16	<2.2e-16
MESO	hsa04142	Lysosome	121	52	-0.61380	-25.980	<2.2e-16	<2.2e-16
OV	hsa04142	Lysosome	121	49	-0.63047	-22.609	<2.2e-16	<2.2e-16
PRAD	hsa04142	Lysosome	121	43	-0.56366	-21.935	<2.2e-16	<2.2e-16
SARC	hsa04142	Lysosome	121	48	-0.58746	-22.827	<2.2e-16	<2.2e-16
SKCM	hsa04142	Lysosome	121	57	-0.62795	-26.360	<2.2e-16	<2.2e-16

STAD	hsa04142	Lysosome	121	52	-0.58374	-22.960	<2.2e-16	<2.2e-16
STES	hsa04142	Lysosome	121	52	-0.56439	-22.075	<2.2e-16	<2.2e-16
TGCT	hsa04142	Lysosome	121	41	-0.46211	-18.455	<2.2e-16	0.0054325
THYM	hsa04142	Lysosome	121	53	-0.55958	-21.679	<2.2e-16	<2.2e-16
UCS	hsa04142	Lysosome	121	54	-0.50815	-23.349	<2.2e-16	<2.2e-16
UCEC	hsa04142	Lysosome	121	54	-0.54030	-22.325	<2.2e-16	<2.2e-16
UVM	hsa04142	Lysosome	121	47	-0.49398	-19.499	<2.2e-16	0.064043
CHOL	hsa04142	Lysosome	121	41	-0.49509	-18.705	<2.2e-16	0.0031701
LIHC	hsa03015	mRNA surveillance pathway	87	35	0.62108	18.428	<2.2e-16	0.00085969
UVM	hsa00510	N-Glycan biosynthesis	48	19	-0.41272	-15.153	<2.2e-16	0.43610
BLCA	hsa04650	Natural killer cell mediated cytotoxicity	122	76	-0.62749	-24.152	<2.2e-16	<2.2e-16
CESC	hsa04650	Natural killer cell mediated cytotoxicity	119	59	-0.57500	-22.750	<2.2e-16	<2.2e-16
HNSC	hsa04650	Natural killer cell mediated cytotoxicity	123	70	-0.51030	-20.871	<2.2e-16	<2.2e-16
SARC	hsa04650	Natural killer cell mediated cytotoxicity	121	56	-0.63585	-26.107	<2.2e-16	<2.2e-16
THCA	hsa04650	Natural killer cell mediated cytotoxicity	123	70	-0.59791	-21.815	<2.2e-16	<2.2e-16

BLCA	hsa04621	NOD-like receptor signaling pathway	163	74	-0.60328	-24.190	<2.2e-16	<2.2e-16
GBM	hsa04621	NOD-like receptor signaling pathway	161	71	-0.62652	-25.040	<2.2e-16	<2.2e-16
HNSC	hsa04621	NOD-like receptor signaling pathway	164	54	-0.54982	-25.239	<2.2e-16	<2.2e-16
OV	hsa04621	NOD-like receptor signaling pathway	161	69	-0.62637	-23.152	<2.2e-16	<2.2e-16
SARC	hsa04621	NOD-like receptor signaling pathway	162	71	-0.61002	-25.966	<2.2e-16	<2.2e-16
THYM	hsa04621	NOD-like receptor signaling pathway	160	44	-0.45002	-17.794	<2.2e-16	0.0094263
UCS	hsa04932	Non-alcoholic fatty liver disease (NAFLD)	143	60	-0.47903	-22.061	<2.2e-16	<2.2e-16
UCEC	hsa04932	Non-alcoholic fatty liver disease (NAFLD)	143	51	-0.47366	-21.517	<2.2e-16	<2.2e-16
UVM	hsa04932	Non-alcoholic fatty liver disease (NAFLD)	142	43	-0.28867	-12.162	<2.2e-16	0.55745
ESCA	hsa04740	Olfactory transduction	371	252	0.52115	14.791	<2.2e-16	0.14126
GBMLGG	hsa04740	Olfactory transduction	371	252	0.52115	14.791	<2.2e-16	0.14126
STAD	hsa04740	Olfactory transduction	404	287	0.42298	14.470	<2.2e-16	0.10147
STES	hsa04740	Olfactory transduction	404	283	0.40958	13.196	0.0040323	0.27108
LAML	hsa04380	Osteoclast differentiation	126	50	-0.67793	-25.000	<2.2e-16	<2.2e-16
LGG	hsa04380	Osteoclast differentiation	126	56	-0.48115	-17.696	<2.2e-16	0.0097584
CESC	hsa04380	Osteoclast differentiation	126	59	-0.60221	-23.970	<2.2e-16	<2.2e-16

ESCA	hsa04380	Osteoclast differentiation	126	62	-0.49123	-24.224	<2.2e-16	<2.2e-16
GBMLGG	hsa04380	Osteoclast differentiation	126	62	-0.49123	-24.224	<2.2e-16	<2.2e-16
KIRP	hsa04380	Osteoclast differentiation	126	59	-0.55470	-21.475	<2.2e-16	<2.2e-16
LUAD	hsa04380	Osteoclast differentiation	126	66	-0.67555	-25.801	<2.2e-16	<2.2e-16
DLBC	hsa04380	Osteoclast differentiation	126	64	-0.54663	-23.905	<2.2e-16	<2.2e-16
PCPG	hsa04380	Osteoclast differentiation	126	49	-0.59126	-21.726	<2.2e-16	<2.2e-16
STAD	hsa04380	Osteoclast differentiation	126	65	-0.75192	-29.745	<2.2e-16	<2.2e-16
STES	hsa04380	Osteoclast differentiation	126	63	-0.74593	-29.791	<2.2e-16	<2.2e-16
THYM	hsa04380	Osteoclast differentiation	126	62	-0.53664	-20.539	<2.2e-16	<2.2e-16
UCS	hsa04380	Osteoclast differentiation	126	54	-0.52029	-25.960	<2.2e-16	<2.2e-16
PCPG	hsa00190	Oxidative phosphorylation	103	38	0.50649	16.572	<2.2e-16	0.17389
KICH	hsa05012	Parkinson disease	114	44	0.39857	14.969	<2.2e-16	0.62234
UVM	hsa05012	Parkinson disease	114	38	-0.38667	-16.850	<2.2e-16	0.22682
UVM	hsa00030	Pentose phosphate pathway	28	8	-0.49582	-16.036	<2.2e-16	0.31808
KIRP	hsa04146	Peroxisome	82	29	-0.61451	-22.711	<2.2e-16	<2.2e-16

PRAD	hsa04146	Peroxisome	82	34	-0.48819	-17.304	<2.2e-16	0.032714
UCEC	hsa04146	Peroxisome	82	30	-0.58949	-22.271	<2.2e-16	<2.2e-16
LGG	hsa04145	Phagosome	145	74	-0.54884	-20.426	<2.2e-16	0.0057204
COADREAD	hsa04145	Phagosome	144	52	-0.50218	-19.125	<2.2e-16	0.0022177
GBM	hsa04145	Phagosome	145	72	-0.63972	-25.264	<2.2e-16	<2.2e-16
KIRC	hsa04145	Phagosome	145	29	-0.39629	-16.615	<2.2e-16	0.051033
KIRP	hsa04145	Phagosome	145	76	-0.59760	-22.195	<2.2e-16	<2.2e-16
DLBC	hsa04145	Phagosome	145	50	-0.51053	-21.772	<2.2e-16	<2.2e-16
MESO	hsa04145	Phagosome	145	82	-0.54370	-22.881	<2.2e-16	<2.2e-16
OV	hsa04145	Phagosome	145	71	-0.69633	-25.668	<2.2e-16	<2.2e-16
PAAD	hsa04145	Phagosome	145	65	-0.49608	-19.480	<2.2e-16	0.00064161
PCPG	hsa04145	Phagosome	144	64	-0.51057	-18.898	<2.2e-16	0.0015315
SARC	hsa04145	Phagosome	145	68	-0.63803	-25.775	<2.2e-16	<2.2e-16
SKCM	hsa04145	Phagosome	145	45	-0.45183	-19.813	<2.2e-16	<2.2e-16
THYM	hsa04145	Phagosome	145	63	-0.54401	-21.431	<2.2e-16	<2.2e-16

UCS	hsa04145	Phagosome	145	73	-0.52871	-24.685	<2.2e-16	<2.2e-16
THCA	hsa04145	Phagosome	145	70	-0.57957	-21.819	<2.2e-16	<2.2e-16
UCEC	hsa04145	Phagosome	145	68	-0.54403	-23.002	<2.2e-16	<2.2e-16
CHOL	hsa04145	Phagosome	144	52	-0.50218	-19.173	<2.2e-16	0.0015850
ESCA	hsa04072	Phospholipase D signaling pathway	140	57	-0.36482	-20.512	<2.2e-16	0.0025706
GBMLGG	hsa04072	Phospholipase D signaling pathway	140	57	-0.36482	-20.512	<2.2e-16	0.0025706
BLCA	hsa04151	PI3K-Akt signaling pathway	349	130	-0.49417	-21.190	<2.2e-16	<2.2e-16
LUSC	hsa04151	PI3K-Akt signaling pathway	350	121	-0.54708	-21.808	<2.2e-16	<2.2e-16
STAD	hsa04151	PI3K-Akt signaling pathway	350	119	-0.46241	-20.584	<2.2e-16	<2.2e-16
ACC	hsa04611	Platelet activation	118	57	-0.56439	-20.602	<2.2e-16	0.0003890 3
KICH	hsa04611	Platelet activation	118	38	-0.42837	-15.736	<2.2e-16	0.12368
PCPG	hsa04611	Platelet activation	118	53	-0.53014	-19.510	<2.2e-16	0.0011643
STES	hsa04611	Platelet activation	118	52	-0.60328	-23.641	<2.2e-16	<2.2e-16
UCS	hsa03050	Proteasome	44	18	-0.56342	-20.330	<2.2e-16	0.0017961
COADRE AD	hsa04974	Protein digestion and absorption	85	43	-0.54888	-19.667	<2.2e-16	0.0013729

TGCT	hsa04974	Protein digestion and absorption	88	50	0.51772	18.152	<2.2e-16	0.034665
CHOL	hsa04974	Protein digestion and absorption	85	43	-0.54888	-19.906	<2.2e-16	0.0012680
COADREAD	hsa04141	Protein processing in endoplasmic reticulum	157	45	-0.47497	-18.434	<2.2e-16	0.0042150
KICH	hsa04141	Protein processing in endoplasmic reticulum	157	53	-0.46341	-17.753	<2.2e-16	0.050891
KIRC	hsa04141	Protein processing in endoplasmic reticulum	159	64	-0.50876	-16.828	<2.2e-16	0.052234
KIRP	hsa04141	Protein processing in endoplasmic reticulum	159	53	-0.55365	-20.302	<2.2e-16	<2.2e-16
DLBC	hsa04141	Protein processing in endoplasmic reticulum	159	61	-0.46064	-19.898	<2.2e-16	0.00045672
KIPAN	hsa04141	Protein processing in endoplasmic reticulum	159	57	-0.53900	-20.013	<2.2e-16	<2.2e-16
PAAD	hsa04141	Protein processing in endoplasmic reticulum	159	62	-0.51626	-20.442	<2.2e-16	<2.2e-16
PCPG	hsa04141	Protein processing in endoplasmic reticulum	157	64	-0.52198	-19.473	<2.2e-16	0.0010534
PRAD	hsa04141	Protein processing in endoplasmic reticulum	159	49	-0.50915	-18.890	<2.2e-16	0.0090187
UCEC	hsa04141	Protein processing in endoplasmic reticulum	159	62	-0.48226	-21.465	<2.2e-16	<2.2e-16
CHOL	hsa04141	Protein processing in endoplasmic reticulum	157	45	-0.47497	-18.803	<2.2e-16	0.0027738
BRCA	hsa05205	Proteoglycans in cancer	196	59	-0.43888	-16.177	<2.2e-16	0.060379
CESC	hsa05205	Proteoglycans in cancer	196	73	-0.47851	-20.355	<2.2e-16	0.00070386

KICH	hsa05205	Proteoglycans in cancer	196	61	-0.41954	-16.553	<2.2e-16	0.086370
KIPAN	hsa05205	Proteoglycans in cancer	196	84	-0.47423	-17.133	<2.2e-16	0.022532
PAAD	hsa05205	Proteoglycans in cancer	196	77	-0.47914	-19.496	<2.2e-16	0.00067935
PCPG	hsa05205	Proteoglycans in cancer	196	61	-0.55047	-21.199	<2.2e-16	<2.2e-16
THCA	hsa05205	Proteoglycans in cancer	196	70	-0.53124	-20.686	<2.2e-16	<2.2e-16
DLBC	hsa00230	Purine metabolism	158	41	0.44012	15.376	<2.2e-16	0.088433
BRCA	hsa00240	Pyrimidine metabolism	96	33	0.52029	16.120	<2.2e-16	0.025875
COADREAD	hsa04015	Rap1 signaling pathway	201	66	-0.45644	-18.237	<2.2e-16	0.0045382
KIPAN	hsa04015	Rap1 signaling pathway	203	65	-0.46618	-17.666	<2.2e-16	0.020234
KICH	hsa04810	Regulation of actin cytoskeleton	199	49	-0.41793	-16.319	<2.2e-16	0.10416
PRAD	hsa04810	Regulation of actin cytoskeleton	208	88	-0.45195	-18.199	<2.2e-16	0.015117
THCA	hsa04810	Regulation of actin cytoskeleton	208	73	-0.56004	-21.474	<2.2e-16	<2.2e-16
KIPAN	hsa04924	Renin secretion	63	34	-0.56463	-18.660	<2.2e-16	0.0077084
SKCM	hsa05323	Rheumatoid arthritis	85	31	-0.54923	-20.611	<2.2e-16	<2.2e-16
ACC	hsa03010	Ribosome	131	63	0.65553	25.671	<2.2e-16	<2.2e-16
BLCA	hsa03010	Ribosome	131	89	0.76611	25.976	<2.2e-16	<2.2e-16

LAML	hsa03010	Ribosome	131	63	0.56801	18.194	<2.2e-16	0.0050076
LGG	hsa03010	Ribosome	131	78	0.79709	24.806	<2.2e-16	<2.2e-16
BRCA	hsa03010	Ribosome	130	74	0.69629	22.244	<2.2e-16	<2.2e-16
COADRE AD	hsa03010	Ribosome	131	71	0.66169	25.638	<2.2e-16	<2.2e-16
GBM	hsa03010	Ribosome	131	94	0.57541	19.213	<2.2e-16	<2.2e-16
HNSC	hsa03010	Ribosome	131	91	0.60689	17.427	<2.2e-16	0.0024247
LIHC	hsa03010	Ribosome	131	75	0.64441	20.133	<2.2e-16	<2.2e-16
LUAD	hsa03010	Ribosome	131	79	0.57893	16.498	<2.2e-16	0.038513
LUSC	hsa03010	Ribosome	131	65	0.47958	17.020	<2.2e-16	0.0091815
DLBC	hsa03010	Ribosome	131	96	0.63774	21.783	<2.2e-16	<2.2e-16
MESO	hsa03010	Ribosome	131	75	0.76371	27.242	<2.2e-16	<2.2e-16
PAAD	hsa03010	Ribosome	131	72	0.54898	17.448	<2.2e-16	0.023845
PRAD	hsa03010	Ribosome	131	89	0.77201	20.730	<2.2e-16	<2.2e-16
SARC	hsa03010	Ribosome	131	72	0.63805	18.330	<2.2e-16	0.0020457
STAD	hsa03010	Ribosome	131	63	0.52385	17.065	<2.2e-16	0.010063
THYM	hsa03010	Ribosome	131	75	0.53119	17.523	<2.2e-16	0.0070006
CHOL	hsa03010	Ribosome	131	71	0.66169	25.946	<2.2e-16	<2.2e-16
LAML	hsa03008	Ribosome biogenesis in eukaryotes	69	35	0.59679	17.506	<2.2e-16	0.0097369
LGG	hsa03008	Ribosome biogenesis in eukaryotes	70	32	0.67715	19.623	<2.2e-16	<2.2e-16
LIHC	hsa03008	Ribosome biogenesis in eukaryotes	70	38	0.58294	16.739	<2.2e-16	0.012179
DLBC	hsa03008	Ribosome biogenesis in eukaryotes	69	42	0.68621	21.486	<2.2e-16	<2.2e-16

OV	hsa03008	Ribosome biogenesis in eukaryotes	70	43	0.48107	15.864	<2.2e-16	0.11403
BLCA	hsa03013	RNA transport	158	58	0.54650	18.926	<2.2e-16	0.0031890
LAML	hsa03013	RNA transport	157	54	0.49474	15.863	<2.2e-16	0.061343
LGG	hsa03013	RNA transport	158	66	0.61212	19.525	<2.2e-16	<2.2e-16
BRCA	hsa03013	RNA transport	158	64	0.58737	19.010	<2.2e-16	0.00017104
ESCA	hsa03013	RNA transport	158	67	0.52542	14.606	<2.2e-16	0.16288
GBM	hsa03013	RNA transport	158	69	0.55930	18.661	<2.2e-16	0.00056799
GBMLGG	hsa03013	RNA transport	158	67	0.52542	14.606	<2.2e-16	0.16288
HNSC	hsa03013	RNA transport	158	47	0.53799	15.915	<2.2e-16	0.025914
LIHC	hsa03013	RNA transport	158	70	0.58661	18.161	<2.2e-16	0.00094028
LUAD	hsa03013	RNA transport	158	66	0.50122	14.525	<2.2e-16	0.17652
LUSC	hsa03013	RNA transport	158	58	0.53098	19.073	<2.2e-16	0.00075353
DLBC	hsa03013	RNA transport	157	59	0.58264	20.234	<2.2e-16	0.00034761
MESO	hsa03013	RNA transport	158	60	0.49204	18.010	<2.2e-16	0.018004
OV	hsa03013	RNA transport	157	64	0.47979	17.486	<2.2e-16	0.031007
STAD	hsa03013	RNA transport	158	67	0.64326	21.160	<2.2e-16	<2.2e-16
STES	hsa03013	RNA transport	158	60	0.64904	19.850	<2.2e-16	<2.2e-16
TGCT	hsa03013	RNA transport	158	53	-0.46252	-19.001	<2.2e-16	0.0038729
THYM	hsa03013	RNA transport	158	43	0.53911	18.514	<2.2e-16	0.0026122
UCEC	hsa03013	RNA transport	158	67	0.50402	15.257	<2.2e-16	0.18565
CHOL	hsa03013	RNA transport	156	44	0.35927	14.178	<2.2e-16	0.21262

SKCM	hsa05132	Salmonella infection	83	24	-0.50620	-19.889	<2.2e-16	<2.2e-16
CESC	hsa04071	Sphingolipid signaling pathway	112	49	-0.50751	-20.704	<2.2e-16	0.00081214
ESCA	hsa04071	Sphingolipid signaling pathway	112	56	-0.53216	-25.250	<2.2e-16	<2.2e-16
GBMLGG	hsa04071	Sphingolipid signaling pathway	112	56	-0.53216	-25.250	<2.2e-16	<2.2e-16
KIRC	hsa04071	Sphingolipid signaling pathway	112	43	-0.50428	-20.332	<2.2e-16	<2.2e-16
ACC	hsa03040	Spliceosome	115	54	0.55246	21.718	<2.2e-16	<2.2e-16
LGG	hsa03040	Spliceosome	115	65	0.72978	22.447	<2.2e-16	<2.2e-16
BRCA	hsa03040	Spliceosome	115	58	0.68636	21.677	<2.2e-16	<2.2e-16
COADREAD	hsa03040	Spliceosome	114	51	0.56416	21.765	<2.2e-16	<2.2e-16
ESCA	hsa03040	Spliceosome	115	56	0.59300	16.301	<2.2e-16	0.035949
GBM	hsa03040	Spliceosome	115	58	0.66317	21.513	<2.2e-16	<2.2e-16
GBMLGG	hsa03040	Spliceosome	115	56	0.59300	16.301	<2.2e-16	0.035949
HNSC	hsa03040	Spliceosome	115	63	0.58863	17.154	<2.2e-16	0.0030915
LIHC	hsa03040	Spliceosome	115	60	0.71541	22.123	<2.2e-16	<2.2e-16
LUAD	hsa03040	Spliceosome	115	49	0.64451	18.484	<2.2e-16	0.0072212
LUSC	hsa03040	Spliceosome	115	59	0.64779	22.100	<2.2e-16	<2.2e-16
DLBC	hsa03040	Spliceosome	114	55	0.59067	19.736	<2.2e-16	0.00026071
MESO	hsa03040	Spliceosome	115	48	0.52803	18.568	<2.2e-16	0.010591
OV	hsa03040	Spliceosome	115	49	0.54887	19.198	<2.2e-16	0.0016319
PAAD	hsa03040	Spliceosome	115	47	0.52501	16.318	<2.2e-16	0.063962

PRAD	hsa03040	Spliceosome	115	52	0.66275	17.897	<2.2e-16	0.0022523
SARC	hsa03040	Spliceosome	115	43	0.51658	14.943	<2.2e-16	0.17389
STAD	hsa03040	Spliceosome	115	67	0.69390	22.290	<2.2e-16	<2.2e-16
STES	hsa03040	Spliceosome	115	54	0.68273	20.723	<2.2e-16	<2.2e-16
THYM	hsa03040	Spliceosome	115	54	0.65481	21.605	<2.2e-16	<2.2e-16
CHOL	hsa03040	Spliceosome	114	51	0.56416	21.500	<2.2e-16	<2.2e-16
PCPG	hsa05150	Staphylococcus aureus infection	52	36	-0.67067	-21.405	<2.2e-16	<2.2e-16
ESCA	hsa05322	Systemic lupus erythematosus	122	59	0.61178	16.742	<2.2e-16	0.033487
GBMLGG	hsa05322	Systemic lupus erythematosus	122	59	0.61178	16.742	<2.2e-16	0.033487
PAAD	hsa05322	Systemic lupus erythematosus	122	50	0.50593	16.039	<2.2e-16	0.075401
TGCT	hsa05322	Systemic lupus erythematosus	122	50	-0.47376	-18.988	<2.2e-16	0.0037279
UCS	hsa04742	Taste transduction	78	44	0.59829	17.417	<2.2e-16	0.024555
UCEC	hsa04742	Taste transduction	80	37	0.60803	17.251	<2.2e-16	0.021375
ESCA	hsa04659	Th17 cell differentiation	105	40	-0.44446	-22.523	<2.2e-16	<2.2e-16
GBMLGG	hsa04659	Th17 cell differentiation	105	40	-0.44446	-22.523	<2.2e-16	<2.2e-16
BLCA	hsa04714	Thermogenesis	189	50	0.44789	15.984	<2.2e-16	0.040676
STES	hsa04668	TNF signaling pathway	110	61	-0.53380	-20.578	<2.2e-16	<2.2e-16
THCA	hsa04668	TNF signaling pathway	110	58	-0.59394	-21.193	<2.2e-16	<2.2e-16
ACC	hsa04620	Toll-like receptor signaling pathway	94	54	-0.55131	-19.310	<2.2e-16	0.00061891

UCS	hsa04620	Toll-like receptor signaling pathway	95	40	-0.47724	-20.698	<2.2e-16	<2.2e-16
LAML	hsa05152	Tuberculosis	172	67	-0.60509	-22.982	<2.2e-16	<2.2e-16
CESC	hsa05152	Tuberculosis	172	66	-0.51602	-22.033	<2.2e-16	<2.2e-16
LUAD	hsa05152	Tuberculosis	174	71	-0.55072	-20.992	<2.2e-16	<2.2e-16
UCS	hsa05152	Tuberculosis	169	66	-0.46315	-21.209	<2.2e-16	<2.2e-16
THCA	hsa05152	Tuberculosis	176	75	-0.53533	-20.398	<2.2e-16	<2.2e-16
KICH	hsa04120	Ubiquitin mediated proteolysis	133	46	-0.44427	-16.696	<2.2e-16	0.11009
TGCT	hsa04120	Ubiquitin mediated proteolysis	133	46	-0.45074	-18.085	<2.2e-16	0.0068345
KIRP	hsa00280	Valine, leucine and isoleucine degradation	47	35	-0.78808	-24.594	<2.2e-16	<2.2e-16
LIHC	hsa00280	Valine, leucine and isoleucine degradation	47	37	-0.85682	-27.540	<2.2e-16	<2.2e-16
SKCM	hsa05416	Viral myocarditis	56	25	-0.48944	-18.129	<2.2e-16	0.030340
SARC	hsa04310	Wnt signaling pathway	143	49	0.53070	15.618	<2.2e-16	0.11251

ES: enrichment score, NES: normalized-enrichment score , FDR: false discovery rate
