# Supplementary figures and images for: Beyond Transposons: TIGD1 as a Pan-Cancer Biomarker and Immune Modulator
Source: Genes (Basel). 2025 May 30;16(6):674. doi: 10.3390/genes16060674 (PMC12192532; doi:10.3390/genes16060674)

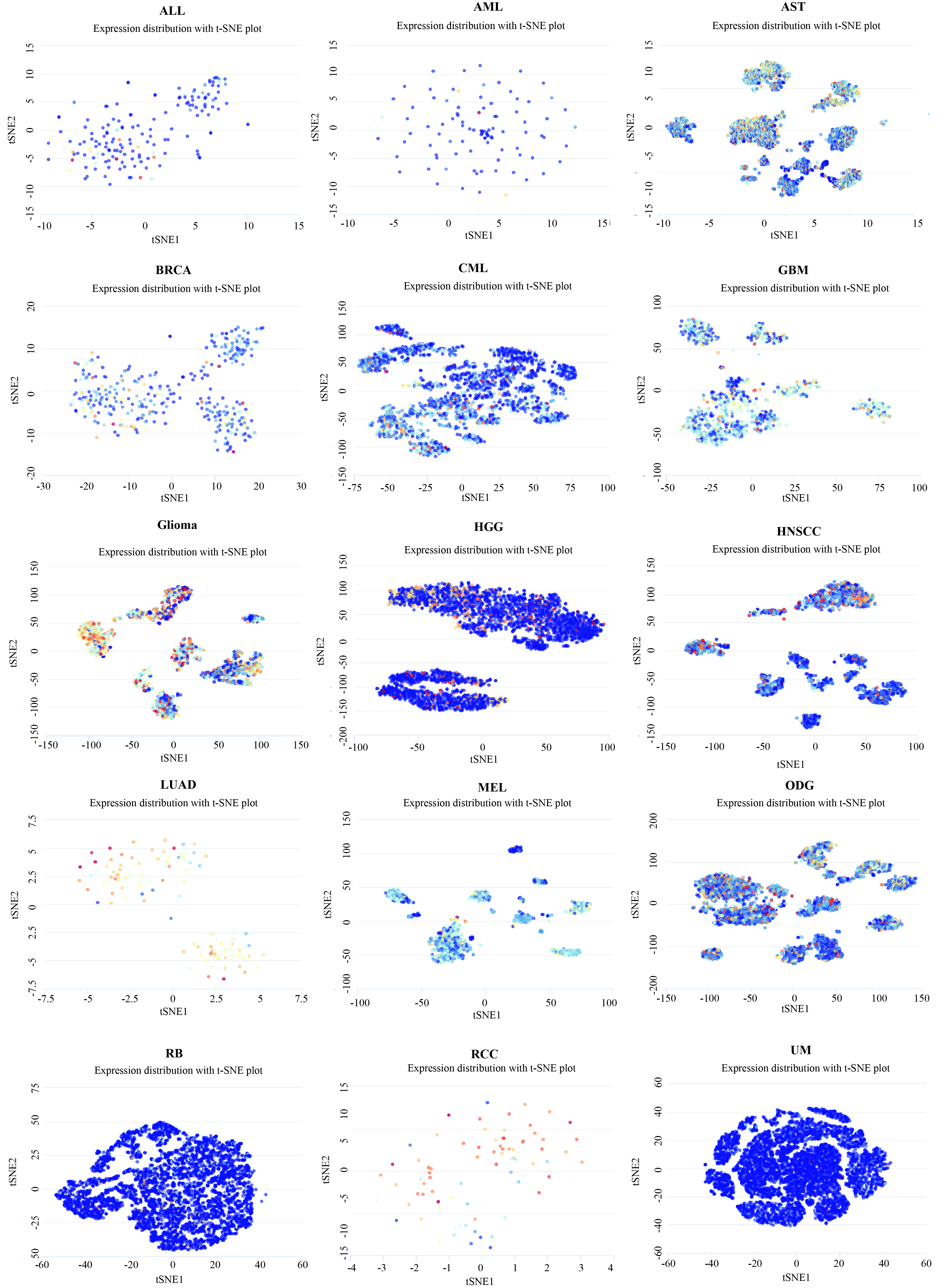

Supplement: Supplementary file 1 [file genes-16-00674-s001.zip › Figure S1.jpg]
